# A charged existence: A century of transmembrane ion transport in plants

**DOI:** 10.1093/plphys/kiad630

**Published:** 2024-01-02

**Authors:** Michael R Blatt

**Affiliations:** Laboratory of Plant Physiology and Biophysics, University of Glasgow, Bower Building, Glasgow G12 8QQ, UK

## Abstract

If the past century marked the birth of membrane transport as a focus for research in plants, the past 50 years has seen the field mature from arcane interest to a central pillar of plant physiology. Ion transport across plant membranes accounts for roughly 30% of the metabolic energy consumed by a plant cell, and it underpins virtually every aspect of plant biology, from mineral nutrition, cell expansion, and development to auxin polarity, fertilization, plant pathogen defense, and senescence. The means to quantify ion flux through individual transporters, even single channel proteins, became widely available as voltage clamp methods expanded from giant algal cells to the fungus *Neurospora crassa* in the 1970s and the cells of angiosperms in the 1980s. Here, I touch briefly on some key aspects of the development of modern electrophysiology with a focus on the guard cells of stomata, now without dispute the premier plant cell model for ion transport and its regulation. Guard cells have proven to be a crucible for many technical and conceptual developments that have since emerged into the mainstream of plant science. Their study continues to provide fundamental insights and carries much importance for the global challenges that face us today.

## Teaser

Membrane transport accounts for one third of energy consumption in plant cells; its study is about growth, development, and survival, not just for the plant but also for the planet.

## Introduction

To posit the past 50 years as marking the transition to modern research on plant membrane transport is understatement. The period encompasses two major revolutions in research, both with a profound impact on our present understanding of plant membranes and their physiology. The first and most obvious is the tectonic shift from traditional biochemical methods to those of molecular genetics. The second, if subtle, is in many ways more profound and is rooted in an awakening within the plant research community to the value of electrophysiology, chiefly of the voltage clamp, in dissecting and quantifying transport. These two drivers have facilitated our understanding of protein structures that support ion flux and its regulation and the molecular mechanics behind subcellular as well as multicellular signal transduction; they underpin the detailed and quantitative understanding we now have of stomatal physiology as well as long-distance signaling in pathogenesis and herbivory; and they are now generating developments in bioengineering that hold real promise for more sustainable ways to live and work within the global environment.

Having researched membrane transport in several model systems over much of this period, I am astounded by the sheer volume of literature now facing anyone starting out, especially those with interests in stomata. When I joined Enid MacRobbie's laboratory in 1983, intent on exploring the electrophysiology of stomatal guard cells, the entire body of literature comprised 4 papers, all empirical observations on guard cell membrane voltage (Pallaghy 1968; Moody and Zeiger 1978; Saftner and Raschke 1981; Ishikawa et al. 1983), and 11 more describing the ion fluxes and contents of guard cells in the closed and open states. Twenty-five years later, in developing the first-generation OnGuard platform to model stomata (Chen et al. 2012; Hills et al. 2012), Adrian Hills, Zhonghua Chen, V.L. (Arieh) Lew, and I sifted through more than 8,000 papers, including a core of almost 500 electrophysiological studies. Integrating the voltage clamp, patch clamp and flux data from these publications allowed us to detail the 23 classes of plasma membrane and endomembrane transporters that contribute directly to osmotic solute and water flux for stomatal movements (Blatt et al. 2022). Five years later, in preparing a major review on the topic for this journal (Jezek and Blatt 2017), Mareike Jezek and I confronted as many publications again. Last year alone saw almost 6,000 publications, roughly 500 per month, incorporating “stomata” and “guard cell” in the title and abstract.

Advances box

- Beginning in the 1980s, adopting voltage clamp and patch clamp methods, plant electrophysiologists established a wealth of quantitative data showing how the dominant pumps, channels, and ion-driven carriers operate in multicellular plants.
- Plant membrane transport is governed by overlapping circuits of H^+^ and charge flux that pass through ion pumps, carriers, and channels. These transporters operate in parallel across each membrane and in series between the plasma membrane and each endomembrane.
- Data for stomatal guard cells, and their relevance to foliar gas exchange and photosynthesis, established these cells as the premier model for studies of membrane transport and its regulation that they are today.
- Membrane ion transport in plants is well-described mathematically with flux equations that incorporate voltage as a driver and parameters resolved for all dominant charge transporters in guard cells.
- Model and experiment show an extraordinary degree of entanglement that gives rise to unexpected, often counterintuitive physiology.

While guard cells are now, without dispute, the premier cell model for studies of membrane transport and its regulation in plants, they have also proven to be a crucible for a much greater breadth of research as a transitional ground, a microcosm of methods and concepts emerging into the mainstream of research in plant membrane transport. I need hardly elaborate, too, that these developments are directly relevant to foliar gas exchange, photosynthesis, and global environmental change (Berry et al. 2010; Smith and Dukes 2013; Lawson and Blatt 2014; Blatt et al. 2022). Stomata play a key role in atmospheric modeling and weather prediction (Beljaars et al. 1996; Betts et al. 1996; Berry et al. 2010; Jasechko et al. 2013), and they are at the center of a crisis in fresh water availability that is presently unfolding (UNESCO 2015; Smedley 2023). It would be impossible to cover more than a tiny fraction of the current knowledge pertinent to membrane transport and its place in plant development, nutrition, signal transduction, stress, and environmental physiology. Here, I have chosen to touch on a small selection of topics that are relevant, both historically and as landmarks going forward. I interweave the first sections of this review with some essential conceptual background to electrophysiology before focusing on what has been, and still remains to be, learned from guard cells.

For the interested reader, I recommend several excellent reviews that cover transport integration, voltage-dependent gating of channels and channel structure, and signaling in guard cells (Lawson and Blatt 2014; Maurel et al. 2015; Assmann and Jegla 2016; Jezek and Blatt 2017; Jegla et al. 2018; Lefoulon 2021; Blatt et al. 2022), Ca^2+^ signaling (Hetherington and Brownlee 2004; McAinsh and Pittman 2009; Roelfsema and Hedrich 2010), action potentials, transport, and signaling in Characean algae (Thiel et al. 1997; Shepherd et al. 2002; Beilby and Al Khazaaly 2016, 2017; Beilby et al. 2022), the Venus flytrap (*Dionaea muscipula*) (Sibaoka 1969; Hedrich et al. 2016), pollen and root hairs (Campanoni and Blatt 2007; Zonia and Munnik 2007), and systemic signaling in pathogenesis and herbivory (Choi et al. 2016; Johns et al. 2021; Klejchova et al. 2021). Readers interested in the conceptual approaches and methods of membrane transport research will find much detail in treatments of the all-important kinetics of transport and its interpretation [see Sanders (1990) and several useful chapters in *Membrane Transport in Plants* (Blatt 2004)], the practical guides *Microelectrode Techniques* (Standen et al. 1987) and *Microelectrode Methods for Intracellular Recording and Electrophoresis* (Purves 1981), as well as the textbooks *Ion Channels of Excitable Membranes* (Hille 2001) and *Cellular Biophysics* (Weiss 1996). An annotated timeline and short history of some of the key developments relevant to plant membrane transport research will be found in Klejchova et al (2021).

## An early focus on membrane voltage

Voltage as a measure of membrane transport is deeply engrained in the collective consciousness of plant physiologists. Prior to the introduction by Cole (1949) of the voltage clamp—a circuit that incorporates the cell membrane in a feedback loop to measure membrane current—voltage was also the principal variable available to the experimenter. Recording membrane voltage required only a high-impedance “follower” amplifier, a set of electrolyte junctions for connection to the cell and bath, and some means to gain electrical access to the inside of a cell. More elaborate circuits to measure membrane conductance by balancing a small stimulus across a Wheatstone bridge required considerable patience and a robust tissue. It is little surprise, then, that membrane voltage was, and in some circles still is, the most commonly cited variable.

Both voltage and conductance measurements appear in the earliest intracellular recordings from plants, predating by two decades similar studies on animal cells (Ling and Gerard 1949; Bretag 2017). The concept of a surrounding cell membrane had been expounded on by Pfeffer in the 1870s (Pfeffer 1877), and a role for the cell membrane in supporting animal “bioelectricity” was recognized early in the 20th century (Bernstein 1902). Yet, it was in plants in the 1920s—around the time of the founding of this journal—that Umrath (1930, 1932), Osterhout (1931), and later Blinks (1942, 1949) were making use of pulled-glass capillaries, microelectrodes filled with salt solutions for electrical contact, in recordings upon insertion into single cells of the giant algae *Chara* and *Nitella* and the marine alga *Valonia* [see also Hope and Walker (1975)]. These cells offered both the size and robustness needed to withstand physical handling and impalements with the relatively coarse microelectrodes of the time, but their use also left doubts at first about whether the findings were applicable to plants more generally. The studies noted a principal dependence on the K^+^ concentration, with voltages largely consistent with passive diffusion of the ion across the cell membrane. They also highlighted the dynamic nature of the membrane, its sensitivity to temperature, and its capacity to support large voltage transients, an “irritability” later identified as action potentials, that accompanied substantial increases in membrane conductance. With the origins of the action potentials in nerves resolved to discrete changes in the conductances for Na^+^ and K^+^ (Hodgkin and Huxley 1952; Hodgkin et al. 1952), what would later be recognized as ion channels, the connection of membrane voltage to passive ionic diffusion was firmly established.

Voltage recordings take on a different, if complementary, perspective as a tool to assess active transport. By convention, biological membranes are referenced to the outside [or, for the tonoplast, to the vacuole volume]. The diffusion of an individual ion is governed by the Nernst Equation:


(1) $$E_{x}=\frac{RT}{zF}\ln\;\left\{\frac{{\left[X\right]}_{o}}{{\left[X\right]}_{i}}\right\},$$

which defines the voltage at equilibrium, *E_x_*, for a single ion *X*. Here, *R* is the universal gas constant, *T* is the temperature in °K, *z* is the charge on ion *X*, and *F* is the Faraday constant. In effect, diffusion of ion *X* progresses until the driving force for diffusion, determined by the ratio of the concentrations inside vs outside, [*X*]_i_/[*X*]_o_, is balanced by an equal opposing electrical driving force, *E_x_*, the equilibrium voltage. The high molar value of the Faraday constant (approx. 10^5^ Coul/mol) and the small electrical capacitance of biological membranes (approx. 10^−6^ Coul/V.cm^2^ = 1 F/cm^2^) means that only a very small number of ions need move across a membrane to reach equilibrium and ensures that this voltage builds up exceedingly rapidly, typically over very small fractions of a millisecond.

For multiple ions, this equation expands, for example in the Goldman–Hodgkin–Katz Equation, to include weightings for the differences in relative conductances (permeabilities) for each ionic species, *p_x_*, so that the resting voltage of the membrane


(2) $$V_{m}=\frac{RT}{F}\ln\;\left\{\frac{p_{K}{\left[K^{+}\right]}_{o}+p_{\mathrm{Na}}{\left[Na^{+}\right]}_{o}+p_{\mathrm{Cl}}{[Cl^{-}]}_{i}}{p_{K}{\left[K^{+}\right]}_{i}+p_{\mathrm{Na}}{\left[Na^{+}\right]}_{i}+p_{\mathrm{Cl}}{\left[Cl^{-}\right]}_{o}}\right\}.$$

Note that *V_m_* is not the same as the ionic equilibrium voltage. Instead, it reflects the balance of ionic fluxes, the voltage at which the sum of all charge movements equals zero. I return to this, most important point shortly.

For a typical plant cell with 150 mM K^+^ and 10 mM Cl^−^ in the cytosol and bathed in a solution of 1 mM KCl outside at 25 °C, the individual equilibrium voltages for K^+^ and Cl^−^ are −126 mV and +58 mV, respectively (Eqn [1]). Provided we can ignore Na^+^, as is often the case for plants, and assuming a realistic conductance ratio *p*_K_*/p*_Cl_ = 10, we can expect a resting voltage near −110 mV (Eqn [2]). At −110 mV, neither K^+^ nor Cl^−^ is at equilibrium, and therefore both ions will slowly diffuse out of the cell, driven towards their respective equilibrium voltages. In most plant cells, these fluxes represent an exceedingly small fraction of the total cellular contents—typically less than 0.000001% s^−1^ for the average mesophyll cell—meaning that diffusion alone may support a charge on a membrane for many hours before the voltage collapses to zero. (Such low conductances also mean that a similarly small increase in conductance for another ion, for example opening channels for Ca^2+^ flux for which E_Ca_ is usually far from the resting voltage, will have a substantial impact on the membrane voltage with very little change in the total ion flux.)

Why are these calculations important? A membrane voltage well in excess (negative) of the equilibrium voltages for all of the major ions present is a good indicator of metabolic energy-dependent ion pumping. So, new evidence that surfaced in the 1960s was a direct challenge to the dogma that the voltages on biological membranes are determined solely by passive ionic diffusion. Clifford and Carolyn Slayman (Slayman and Slayman 1962; Slayman 1965) reported membrane voltages from the fungus *Neurospora* of −200 mV, far beyond the most negative equilibrium voltage for K^+^ of −25 mV. Most importantly, these studies also showed that the membrane collapsed to voltages near K^+^ equilibrium when respiration was inhibited by treatment with azide, dinitrophenol or carbon monoxide. Once it was recognized that small amounts of Ca^2+^ were important to reduce so-called leak conductances, similarly large, inside-negative voltages were reported in plant cells as well (see Spanswick 1981).

Identifying the ion primarily responsible for the membrane voltage is often a process of elimination. The discovery (Kitasato 1968) that the membrane voltage of *Nitella* is strongly dependent on pH and often substantially more negative than all other ionic equilibria was a vital breakthrough; it helped establish the concept of plasma membrane ATPases that pump H^+^ out of the cell, with a major proportion of the energy of ATP hydrolysis used to maintain a large transmembrane voltage. The concept of H^+^ as the driver ion coupling the “economy” of transport in plants and fungi gained support in the 1970s and 1980s in the wake of Peter Mitchell's ideas of chemiosmosis (Mitchell 1969). Studies of *Neurospora* (Slayman and Slayman 1974; Hansen and Slayman 1978; Blatt et al. 1997) and of microalgae (Schwab and Komor 1978), liverworts (Felle 1981), Characean algae (Beilby and Walker 1981; Sanders and Hansen 1981; Sanders et al. 1985), and angiosperm plants (Ullrich et al. 1984; Basso and Ullrich-Eberius 1987; McCutcheon and Bown 1987; Meharg and Blatt 1995) showed that the membrane voltage depolarized and the extracellular medium alkalinized when uncharged or negatively charged solutes such as glucose, Cl^−^, NO^−^, and glutamate were taken up, for the anions against the voltage across the membrane. These observations could only be explained if these solutes were transported together with an inward flux of H^+^. The findings left no doubt that transport in plant and fungal cells is coupled to the H^+^ electrochemical gradient, with the H^+^-ATPase generating an electrochemical driving force for H^+^ that is then used to drive the coupled transport of other solutes; they also established the H^+^-ATPase, and by inference membrane transport generally, as a major consumer of metabolic energy, some 30% to 35% in most estimates [see Klejchova et al. (2021), Jezek and Blatt (2017), and Sanders and Slayman (1982)].

So, is membrane voltage a good indicator of H^+^-ATPase activity? The answer is a qualified “yes”. When compared with ionic diffusion equilibria, membrane voltage is useful as a “first test” for energy-dependent transport. However, as a readout with which to quantify pump activity, voltage is a problematic measure. Not least, the observations that plant and fungal membranes depolarize in the presence of uncharged and negatively charged solutes clearly demonstrate that voltage reflects the activities of more than just ATP-dependent H^+^ pumps (see Current matters, below).

## Some pitfalls of the voltage readout

Of course, a major difficulty in quantifying H^+^ flux across any membrane is simply that the H^+^ is highly buffered through its association and dissociation with H_2_O, which is present in aqueous solution at a concentration of roughly 55 mol/L. Therefore, undertaking unidirectional flux measurements is virtually impossible. Yet relying on voltage alone as a readout for pump activity—like net H^+^ (pH)—can lead to serious errors of interpretation. Evidence surrounding the concept of the H^+^–K^+^ exchange ATPase offer a prime example of the danger of relying on voltage as a measure of pump activity. The solution to this problem lies in the use of membrane current, rather than voltage, as a measure of transport. Its resolution was my first major venture into membrane physiology as I started out at the Yale University Medical School in 1980 as a postdoctoral researcher with Clifford Slayman.

When replete with K^+^ (and in the presence of small amounts of Ca^2+^), plant and fungal cells show membrane voltages that are highly sensitive to extracellular H^+^ concentrations in the micromolar and submicromolar range (pH 5 to 7) but change very little when extracellular K^+^ concentrations are reduced below 1 mM [cf. Spanswick et al. (1967), Blatt (1987b), Beilby and Blatt (1986)]. This H^+^ dependence indicates a substantial conductance to H^+^ and, as noted above, was a key piece of evidence for the presence of ATP-powered H^+^ transport. However, when starved of K^+^ so that the cellular K^+^ content falls below approximately 60 mM, the membrane voltages of the same cells become hyperpolarized and strongly dependent on extracellular K^+^ concentration in the micromolar range. For example, Rodriguez-Navarrro et al. (1986) reported that the membrane voltage of K^+^-starved *Neurospora* in the absence of K^+^ was typically near −300 mV; adding even 5 µM K^+^ outside depolarized the membrane by +70 mV; and K^+^ uptake occurred in near one-to-one exchange with H^+^. These findings complemented earlier studies of K^+^-starved yeast that, likewise, showed acidification of the medium that was stimulated by K^+^ additions outside in apparent exchange of H^+^ with K^+^ (Conway and O’Malley 1944; Conway and Brady 1950).

**Figure 1.**
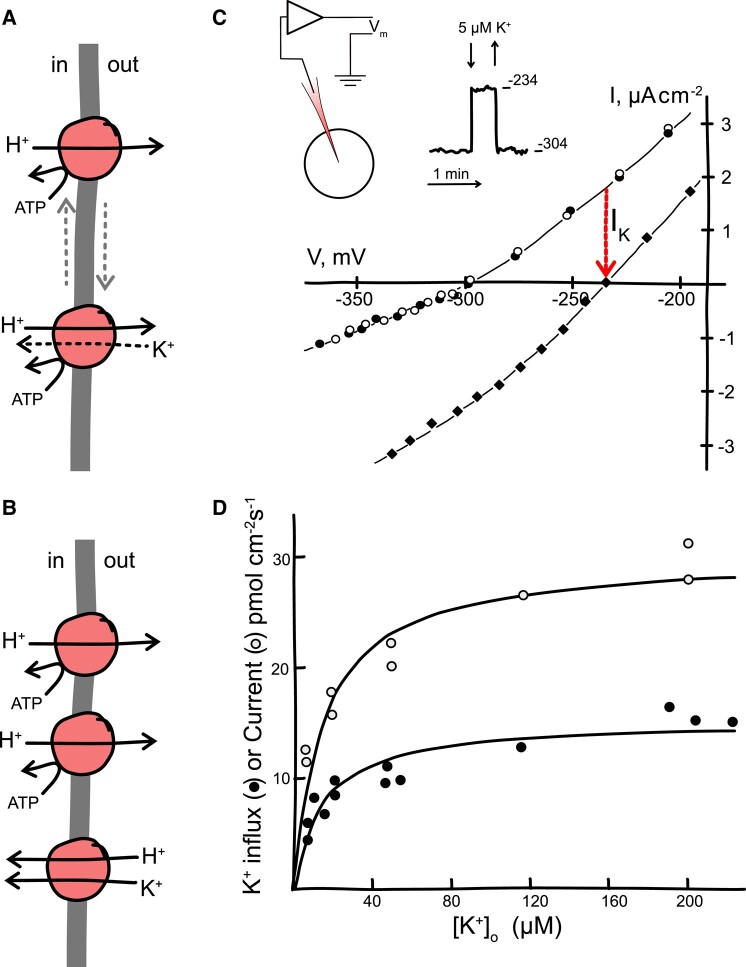
Alternative models for high-affinity K^+^ transport and data critical for selecting between them. **A)** The H^+^/K^+^-ATPase “slippage” model effectively postulated two interchangeable modes of operation for the pump: one that hydrolyses ATP to transport H^+^ out of the cell (*above*) and contributes to membrane voltage, and the second that additionally transports H^+^ out of the cell in exchange for K^+^ uptake (*below*). The second mode was proposed to engage on K^+^ starvation and to operate concurrently with the first mode in order to explain membrane hyperpolarization. **B)** The chemiosmotic model proposes that the H^+^-ATPase (*above*) and a separate H^+^–K^+^ symporter (*below*) are coupled across a common membrane. Disabling the symporter by removing K^+^ outside would cause the H^+^-ATPase to hyperpolarise the membrane toward its equilibrium voltage, offset only by a small background conductance (not shown). In the presence of K^+^ outside, the membrane would depolarize, with the current through the H^+^-ATPase balanced by the symporter so that two H^+^ ions would pass through the H^+^-ATPase for every pair of charges, one H^+^ and one K^+^, returning through the symporter. **C)** Current–voltage (IV) curves recorded from K^+^-starved *Neurospora* before (❍), upon adding 5 µM K^+^ (♦), and after removing K^+^ from the bath (●). The current introduced at the free-running voltage on adding K^+^ (I_K_) is indicated (red arrow). *Insets*: Simplified circuit for recordings from spherical cells of *Neurospora* and the free-running voltage before, during, and after adding 5 µM K^+^ (free-running voltages and time scale indicated by trace). The simplified circuit comprises an amplifier (triangle) that reports the membrane voltage (*V*_m_) from electrode impaled in the spherical cell (circle) referenced to the earthed bath solution. **D)** Net chemical flux (^42^K^+^, ●) and current at the free-running voltage (❍) determined from concurrent radiotracer and voltage clamp recordings of *Neurospora* such as shown in **(C)**. Current here converted to units of pmol cm^−2^ s^−1^ for direct comparison with chemical flux. The results show that two charges move across the membrane with each K^+^ ion. Data in **(C)** and **(D)** are redrawn from Rodriguez-Navarro et al. (1986).

Why should K^+^ starvation lead to large (negative) membrane voltages, and why should adding micromolar K^+^ outside evoke such large voltage changes with an exchange of K^+^ uptake with H^+^ release? One interpretation (Fig. 1A) was that the large voltages were a consequence of enhanced ATPase activity, and the stimulation by K^+^ represented a mode of pump operation that, in K^+^-starved cells, engages an alternative transport cycle to couple K^+^ uptake in exchange with H^+^. This concept of a H^+^/K^+^-ATPase was heavily influenced by contemporary studies of mammalian Na^+^/K^+^-ATPases and was proposed to incorporate “slippage” that would accommodate electrogenesis as well as H^+^ exchange with K^+^, subject to the K^+^ content within the cell. These ideas, and suggestive biochemical studies with microsomal membrane vesicles (Hodges et al. 1972; Leonard et al. 1973), led to more than a decade of research beginning in the 1970s in an ultimately futile search for this hypothetical plant H^+^/K^+^ exchange ATPase (Leonard and Hotchkiss 1976; Briskin and Leonard 1982). [As late as the 1980s, the concept that membrane voltages, plant or animal, could be determined by more than ionic diffusion was ridiculed in some circles. The electrogenic nature of the mammalian Na^+^/K^+^-ATPase, itself, remained a matter of controversy for over 30 years and ended only following studies in the mid-1980s that demonstrated a current carried by the Na^+^/K^+^-ATPase (DeWeer et al. 1988)].

An alternative explanation for the extreme negative voltages and sensitivity to K^+^ outside is that K^+^ starvation promotes the expression of transporters that achieve a high affinity for K^+^ by coupling its uptake, in symport, to the influx of H^+^ (Fig. 1B). Physical laws dictate that in the steady state, the net charge movement across a membrane must always be zero at the free-running voltage, a point I noted earlier. So, such a transporter, together with a H^+^-ATPase, would explain the observations, provided that H^+^ flux through the ATPase was balanced primarily by charge return through H^+^-coupled K^+^ transport: in other words, for every two H^+^ ions pumped out of the cell by the H^+^-ATPase, two charges, one H^+^ and one K^+^, would enter the cell. The overall balance sheet, then, would give a net exchange of H^+^ with K^+^, acidification of the external medium, a strongly rheogenic uptake of K^+^ with two charges moved with each K^+^ and, with K^+^ outside removed to disable the H^+^-K^+^ return pathway, an extremely negative membrane voltage driven by the H^+^-ATPase.

The idea of H^+^-coupled cation symport had been mooted in bacteria (Bakker and Harold 1980) but met with stiff resistance: in the early 1980s, cation uptake by eukaryotes was universally assumed to be driven by voltage only, negative inside the cell, or directly by ATP hydrolysis as is the case for K^+^ in animal and insect cells. So, the finding by Rodriguez-Navarro et al (1986) that high-affinity K^+^ uptake in K^+^-starved *Neurospora* occurred in 1:1 exchange with H^+^—and, most telling, that K^+^ additions depolarized the membrane—was a direct challenge to this dogma (Fig. 1C). Comparisons of ^42^K^+^ radiotracer flux with K^+^ current showed that two charges enter the cell with each K^+^ ion (Fig. 1D), precluding any notion of a channel-mediated K^+^ flux (Hedrich and Schroeder 1989). Instead, the instantaneous and reversible depolarizations upon adding K^+^ outside indicated that a strongly rheogenic transporter operated in a chemiosmotic cycle with the H^+^-ATPase. This interpretation also explained why uptake was inhibited by metabolic blockade to deplete the energy supply.

Ultimately, characterizing any transporter—whether a pump, ion-driven carrier or channel—requires the transport rate to be quantified as a function of the relevant substrate(s). Current is a direct measure of transport rate, whereas voltage is not. Furthermore, for any transporter that moves charge and is therefore subject to membrane voltage, control of the voltage is essential for quantification. The voltage clamp provides the means to this control, and it yields the membrane current associated with each clamp voltage that defines the current-voltage (IV) relation of the membrane. Of course, every cell harbors a number of different transporters that move a net charge across the membrane and will therefore contribute to the total membrane current. So, the challenge for the experimenter is to isolate that fraction of the current carried by the transporter of interest.

One common approach to this challenge is to use an inhibitor to selectively block the transporter and record membrane current at each voltage before, and immediately after, adding the inhibitor. The difference between the two sets of measurements can then be credited to the transporter of interest. Good examples of this approach is the use of Ba^2+^ and the tetraethylammonium cation to block K^+^ channels (Hagiwara et al. 1978; Blatt 1988b; Tester 1988; Wegner et al. 1994; Blatt and Gradmann 1997) and of ouabain to inhibit the Na^+^/K^+^-ATPase (Skou and Norby 1979; Karlish and Stein 1982). In the absence of a selective inhibitor, transport may be blocked by removing a substrate. In the case of high-affinity K^+^ transport, for which there is no specific inhibitor, transport can be suppressed by removing K^+^ outside. (In practice, K^+^ leaching from glass puts a lower limit on [K^+^]_o_ in distilled water of approx. 0.2 µM, but this concentration is almost two orders of magnitude below the apparent K_1/2_ for high-affinity K^+^ uptake.) A difficulty with any approach that alters a substrate concentration is that it affects the thermodynamic driving force on the transporter so that current subtractions become distorted near and beyond the equilibrium voltage. However, current subtractions will yield identifiable subsets of relations, so-called difference current-voltage curves (dIVs), that define the limiting transport kinetics and even the (hidden) equilibrium characteristics (Blatt 1986).

For *Neurospora*, the dIVs for high-affinity K^+^ uptake showed a strong voltage dependence with the difference current saturating at voltages negative of −250 mV. Using the voltage clamp to control voltage, it was possible to demonstrate that the K^+^ current was independent of H^+^-ATPase activity and not directly affected by metabolic blockade; in the absence of the voltage clamp, K^+^ uptake was inhibited by metabolic blockade, but only because inhibiting the H^+^-ATPase led to large depolarizations of the membrane (Blatt et al. 1987; Blatt and Slayman 1987). A decade later, work with purified maize (*Zea mays*) plasma membrane vesicles ruled out a direct stimulation by K^+^ of the H^+^-ATPase (Gibrat et al. 1990) and drew to a close the hunt for a H^+^/K^+^-ATPase in plants. Subsequent voltage clamp studies of K^+^-starved Arabidopsis (*Arabidopsis thaliana*) roots uncovered evidence for a H^+^–K^+^ symporter (Maathuis and Sanders 1994) much like that of *Neurospora*.

These high-affinity K^+^ transporters are now identified as members of the widely expressed KT/KUP/HAK family that support K^+^ nutrition, growth, and stress responses (Quintero and Blatt 1997; Ahn et al. 2004; Osakabe et al. 2013; Véry et al. 2014). Nevertheless, the physiological and kinetic studies with *Neurospora* stand as a landmark in demonstrating a true chemiosmotic cycle that connects high-affinity, H^+^-coupled K^+^ uptake with the H^+^-ATPase at the plasma membrane, and they underline the importance of voltage as a common intermediate shared between the two transporters. Much the same characteristics are now known for the H^+^-ATPase of *Chara* (Blatt et al. 1990a) and guard cells (Blatt 1987a; Lohse and Hedrich 1992), and their voltage sensitivities are a key feature defining cellular transport in plants.

## Current matters

Why is current important and how does it relate to membrane voltage? Quite simply, transport current is a stoichiometric readout of transport rate and therefore of the kinetic properties of an ion-transporting enzyme. Voltage is both a driving force on transport—the electrical analog of a “substrate” for a transporter that moves charge—and a product of charge movement across the membrane. For high-affinity K^+^ uptake by fungi and plants, voltage is a product of the H^+^-ATPase and a substrate for K^+^ uptake by the H^+^–K^+^ symporter. Of course, the difference here between voltage as a substrate and as a product is a matter of perspective: voltage will act on the charge flux as a “substrate”, and the charge flux in turn will affect the membrane voltage as a “product”. For example, the plasma membrane H^+^-ATPase uses ATP hydrolysis to pump H^+^ out of the cell, thereby contributing to a voltage “product” that is negative inside the cell. At the same time, this voltage also acts as a driver “substrate” that reduces H^+^ efflux via the H^+^-ATPase by opposing the movement of H^+^ out of the cell. Not surprisingly, the plasma membrane H^+^-ATPases of fungi and plant cells show a significant dependence on voltage across the physiological voltage range (Gradmann et al. 1978; Sanders et al. 1981; Blatt 1987a; Blatt and Slayman 1987; Blatt et al. 1990a; Chen et al. 2012).

**Figure 2.**
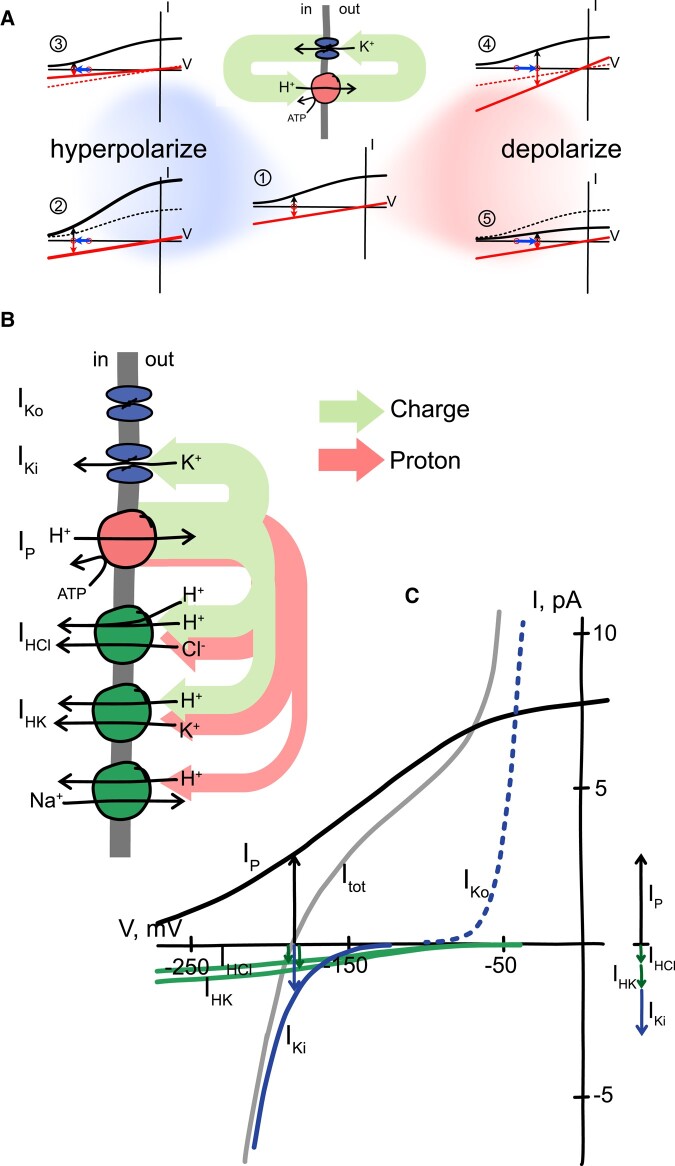
The charge and ionic circuits typical of plant membranes. **A)** A minimal charge circuit for a plant membrane, incorporating a H^+^-ATPase and K^+^ channel, illustrates the codependence of the two arms of the current cycle, outward via the H^+^-ATPase and inward via the channel (*central schematic and green arrows*), and their impacts on membrane voltage. In the steady state, charge movement (current) through the two arms must always be equal in amplitude and opposite in direction across the membrane (vertical *black* and *red arrows*). Thus, from the central current–voltage (IV) plot (1), membrane hyperpolarization is possible (2) by increasing the H^+^-ATPase activity (*black curve*), and/or (3) by decreasing the activity of the current return pathway through the K^+^ channel (*red curve*). Conversely, membrane depolarization is possible (4) by increasing the activity of the current return pathway through the K^+^ channel and/or (5) by decreasing the H^+^-ATPase activity. The initial and final free-running voltages in each case is marked by a red circle and the shift in voltage marked by horizontal blue arrows in each plot. Previous H^+^-ATPase and K^+^ channel activities from the central plot are shown as dotted lines in each case. **B)** Simplified charge and chemical (H^+^) circuits of the plasma membrane illustrated with arrows weighted to show the overall balance of chemical (H^+^) and charge flux. A similar pair of circuits occurs at the tonoplast. Physical laws require that the net charge flux (*green arrows*) across the membrane in the steady state must sum to zero. [Note: The chemical (H^+^, *red arrows*) circuit does not need to balance in the same way.] In other words, at the free-running membrane voltage charge passing out of the cell, here shown as charge movement through the H^+^-ATPase (I_P_), must be the same as the sum of charges passing back into the cell through the other transporters (active charge-carrying transport, I_HCl_, I_Ki_, I_HK_). As a consequence, a change in charge flux through any one transport pathway will affect the balance of charge movement through all of the other transporters that move charge. Note that one-to-one exchange transport (antiport) of a positively charged solute with H^+^, here shown as a Na^+^/H^+^ antiporter (Quintero et al. 2000; Pardo et al. 2006; Bassil and Blumwald 2014), does not result in net charge movement across the membrane and therefore does not contribute to charge balance. **C)** The simplified charge circuit of the plasma membrane in **(B)** illustrated as current–voltage (IV) curves for each of the transporters to show the *capacity* for current through each transporter as a function of voltage. Total membrane current (I_tot_) comprises the sum of the transporter currents, here the H^+^-ATPase (I_P_), the H^+^–K^+^ (I_HK_), and H^+^–Cl^−^ (I_HCl_) symports, and the inward- and outward-rectifying K^+^ channels (I_Ki_ and I_Ko_). At the free-running voltage, the point at which I_tot_ crosses the voltage axis, the vector sum of these currents is zero (*right*). Note that the outward-rectifying K^+^ channels contribute to I_tot_ but, because of their gating properties, only at voltages well positive of the free-running voltage.

An equally important, if more subtle, aspect of these relations pertains to voltage as an intermediate shared between all charge-carrying transporters in a membrane. As noted above, basic laws of physics dictate that net charge movement across a membrane must always sum to zero in the steady state. [The speed at which biological membranes charge, as noted earlier, ensures that a steady state is virtually always maintained. Even during the voltage transient of an action potential, the net charge movement across the membrane remains essentially zero, millisecond by millisecond, as charging of the membrane requires only a negligible fraction of the ion flux (Thiel 1995).] So, the charge (ion) flux through each transporter is conjoined to the flux through all other transporters that carry net charge and affect—and are affected by—voltage across the same membrane. An immediate consequence is that changes in voltage, for example membrane hyperpolarization, may arise either from an increase in H^+^-ATPase activity or from a decrease in the activity of the balancing current return pathways (Fig. 2). Only by clamping the voltage is this interconnection between transporters bypassed, enabling the experimenter to characterize individual transporters using manipulations such as those I used to isolate the *Neurspora* H^+^-K^+^ symporter (Blatt et al. 1987; Blatt and Slayman 1987) and the H^+^–NO_3_^−^ symporter in Arabidopsis roots (Meharg and Blatt 1995). In other words, without the voltage clamp, changes to any charge-carrying transporter will always influence the activities of other transporters in the same membrane.

The position of membrane voltage as a common intermediate often has consequences that are unexpected, even counterintuitive. One example serves to illustrate this point. The *open stomata2* (*ost2*) mutation in Arabidopsis (Merlot et al. 2007) affects the predominant H^+^-ATPase AHA1, rendering it insensitive to Ca^2+^. It promotes membrane hyperpolarization and reduces the dynamic range of stomatal movements. As an intuitive assessment, these observations would suggest that H^+^-ATPase activation simply prevents full stomatal closure in the dark or in abscisic acid (ABA), a water-stress signal, by maintaining membrane energization. It was to be expected that stomata of the *ost2* mutant would close more slowly than wild-type stomata. Counterintuitively, however, stomata of the *ost2* mutant also opened more slowly than the wild type. This observation could be explained by the finding that, under voltage clamp, the inward-rectifying K^+^ current—the KAT1 K^+^ channel activity—in the *ost2* mutant was reduced to 10% or less of the currents recorded in wild-type guard cells (Wang et al. 2017). In other words, the *capacity* for K^+^ flux was greatly reduced (see also Modeling guard cells, below).

Why should a mutation that uncouples the control of the H^+^-ATPase and promotes its activity slow stomatal opening as well as closing, and why should it greatly reduce the K^+^ channel current? The answer lies in the connections to membrane voltage as a shared intermediate between the H^+^-ATPase and the R-type anion channels on one hand, and their connections to plasma membrane Ca^2+^ channels and, hence, to cytosolic-free [Ca^2+^] ([Ca^2+^]_i_) and cytosolic pH on the other. Wang et al (2017) found that the *ost2* mutation, by uncoupling the control of the H^+^-ATPase, elevated the resting [Ca^2+^]_i_ and cytosolic pH. Both the rise in [Ca^2+^]_i_ and in pH suppressed the K^+^ current to slow opening; and, by hyperpolarizing the membrane and raising pH, the effect was also to suppress activation of the R-type channels (Meyer et al. 2010). The latter are a key to driving [Ca^2+^]_i_ and voltage oscillations (Grabov and Blatt 1997, 1998, 1999; Hamilton et al. 2000) that accelerate stomatal closure (Hamilton et al. 2000; Garcia-Mata et al. 2003; Blatt et al. 2007; Minguet-Parramona et al. 2016).

There are several insights to be gained from this study. The first and most obvious is of voltage as a shared intermediate, in this case shared between the H^+^-ATPase and Ca^2+^ channels that trigger [Ca^2+^]_i_ elevations as well as the K^+^ and anion channels. These connections, and changes in the cytosolic H^+^ concentration that are expected of H^+^-ATPase stimulation, have far-reaching consequences, affecting—and connecting—virtually all of the major transporters localized to both the plasma membrane and tonoplast (Jezek and Blatt 2017; Vialet-Chabrand et al. 2017; Klejchova et al. 2021). Furthermore, as transport processes at these two membranes also share a common pool of solutes within the cytosol, transport across one membrane will also influence, and be influenced by, transport across the other (Horaruang et al. 2020), and transport at both membranes will be affected by metabolism (Santelia and Lawson 2016; Jezek and Blatt 2017). As a corollary here, an all-important message is that intuition is often a very poor guide to understanding the physiological impacts of transport and to predicting its behaviors, a point I return to later.

## Ion channels and the patch clamp

“A channel is a hole with a very special role” begins the rhyming couplet. Indeed, so special were they viewed that, for a time, ion channels were widely associated with the Characean algae, cells that had come to be synonymous with action potentials in plants. Equally, it was commonly held that channels were absent from most other plant cells, none of which were thought to feature action potentials. Beginning in the mid-1980s, two separate developments closed any debate and would also shift the focus of much of plant transport research for the next decades to address channel activities and their functions.

The first of these developments was the demonstration of single-channel activities in protoplasts of guard cells from the broad bean *Vicia faba* and the mesophyll of wheat, *Triticum aestivum*. Influenced by work with mammalian cells, Schroeder et al (1984) restricted their recordings across a narrow voltage range and to short time intervals, leading to the erroneous conclusions that the K^+^ channels of these cells were insensitive to membrane voltage and that their activity did not vary with time. Moran et al (1984) observed a range of discrete, step-like currents in leaf mesophyll protoplasts of wheat, including a conductance of 35 pS with a voltage-dependence to its activity, but did not identify the ions transported. Nonetheless, the findings of quantized activities of single channels in the plasma membranes of wheat leaf cells and *Vicia* guard cells left no doubt about their broader presence within the cells of plants. By the beginning of the 1990s, a number of K^+^, Cl^−^, and anion-permeable channels were documented in the plasma membranes of marine algae (Bertl et al. 1988), several cell types of land plants (Schroeder et al. 1987; Bush et al. 1988; Hosoi et al. 1988; Moran et al. 1988; Stoeckel and Takeda 1989; Colombo and Cerana 1991; Spalding et al. 1992), and also in mitochondria (Smack and Colombini 1985), chloroplasts (Schoenknecht et al. 1988; Tester and Blatt 1989), and isolated vacuoles (Hedrich et al. 1986; Hedrich and Kurkdjian 1988; Alexandre and Lassalles 1990; Smith et al. 1990).

The second of the developments stems from the gradual recognition that action potentials—and, by inference, the excitabilities unique to ion channels—are a common feature of most, if not all plant cells, not just of the Characean algae, the traps of insectivorous plants, and pulvinar cells of *Mimosa* and its relatives (Sibaoka 1969; Williams and Pickard 1972a, 1972b; Simons 1981). Electrical excitability with bona fide action potentials were described early on in *Neurospora* (Slayman et al. 1976) and subsequently in the leaves of the marine angiosperm *Zostera* (Garrill et al. 1994), in the vasculature of pea, Arabidopsis, barley, and maize (Fromm and Bauer 1994; Herde et al. 1999; Favre et al. 2001; Felle and Zimmermann 2007), and of course in guard cells (Thiel et al. 1992; Gradmann et al. 1993; Blatt and Thiel 1994; Minguet-Parramona et al. 2016).

Gerhard Thiel recorded the original cardiac-like action potentials of guard cells. To the best of my knowledge, these are also the earliest recordings from the cells of any land plant beyond those of the traps of the insectivorous plant *Dionea muscipula*. Gerhard arrived in Cambridge in 1988 as a postdoctoral researcher with Enid MacRobbie. His unorthodox and eclectic approach was the perfect anodyne to the regimented focus my research had followed until then, and we were soon fast friends. Together we demonstrated inositol trisphosphate (IP_3_)-mediated control of guard cell K^+^ channels via endomembrane Ca^2+^ release (Blatt et al. 1990b). Later, we flagged the puzzle that remains of how a peptide of the auxin-binding protein ABP1 triggers large and near-instantaneous cytosolic alkalinizations that also regulate these K^+^ channels (Thiel et al. 1993a). While at Cambridge, Gerhard went on to resolve the single-channel gating characteristics of the anion channels behind the *Chara* action potential (Thiel et al. 1993b, 1997). These studies firmly established the sequence of their activation by [Ca^2+^]_i_ in what is now the model for Ca^2+^-induced Ca^2+^ release that underpins the action potentials and osmotic K^+^ and Cl^−^ release in plant cells.

Many of the early studies of ion channels in land plants took advantage of the new patch clamp methods devised by Neher and Sakmann (1976) and later refined with improvements in pipette manufacture (Hamill et al. 1981). In the so-called whole-cell configuration, the patch clamp yields data equivalent to those of the voltage clamp. Like the comparator at the heart of the voltage clamp circuit, the patch clamp is a current-to-voltage converter. The patch clamp differs from that of the voltage clamp principally in its omission of a follower amplifier input. For the experimenter, therefore, the patch clamp is always engaged to clamp the membrane to a user-specified voltage (Sigworth 1983). The comparator of the voltage clamp, by contrast, can be bypassed during recordings, allowing the user to record the true free-running voltage across the membrane with the follower amplifier. Nonetheless, patch clamp methods offered access to virtually any cell type in the plant through the expedient of protoplast generation. It circumvented the problems associated with current spread in intact plant tissues that I come to shortly. Exceptions to the patch clamp approach were the use of vesicle fractions fused into planar lipid bilayers to study endomembrane channels, including K^+^ channels of chloroplast thylakoids (Tester and Blatt 1989), and my own work on guard cells that drew on the classical, two-electrode voltage clamp (Blatt 1988b, 1990, 1992; Blatt and Clint 1989; Blatt et al. 1990b).

The patch clamp offers other advantages as well, but it also presents certain limitations in studies of ion channels. Among the advantages, the most important is that, in single-channel configuration, the patch clamp enables analysis of the molecular kinetics of protein conformations that determine gating. These details are resolved by analyzing the open and closed lifetime distributions, in other words the time of residence of the channel in each state. Indeed, recordings of single channels in membrane patches are still the only example in which the molecular dynamics of an individual protein molecule can be studied in situ and in real time. Very few researchers have availed themselves of this facility, however, and even today there remain barely a handful of publications that describe the lifetime kinetics of any plant ion channels. Notable are studies of the gating of the KAT1 K^+^ channel (Hoshi 1995; Zei and Aldrich 1998; Tang and Hoshi 1999) and its regulation by binding to vesicle-trafficking proteins (Lefoulon et al. 2018), of the stelar SKOR K^+^ channel and the K^+^ dependence of its gating (Johansson et al. 2006), and of how Ca^2+^ regulates the activity of Ca^+^ channels at the guard cell plasma membrane (Hamilton et al. 2000, 2001).

Because patch pipettes are usually prepared with tip diameters of 1 µm or slightly greater, diffusion from the pipette allows rapid dialysis of the cell contents against the much larger volume of the patch pipette. In effect, dialysis with the pipette solution allows for experimental control of the cytosolic contents. However, such dialysis also implies that mechanisms of channel regulation that depend on local and transient changes in vivo, for example in [Ca^2+^]_i_, will be masked and can be difficult to assess. A related limitation of patch methods is that dialysis against the pipette often leads to so-called rundown in channel activity as cofactors, essential kinases, and phosphatases, and other regulatory proteins are diluted and lost (Tang and Hoshi 1999; Hamilton et al. 2000; Horvath et al. 2002).

## Voltage clamp and the intact cell

Voltage clamp methods with intracellular microelectrodes avoid the need to generate and maintain protoplasts. They also minimize the problems of cellular dialysis. The tip diameters of microelectrodes are commonly near 0.05 µm and lead to diffusion rates typically 300- to 1000-fold less than those of patch pipettes (Blatt and Slayman 1983; Sakmann and Neher 1983; Blatt 1987b). However, work with intact tissues presents a different set of constraints for the electrophysiologist. Of these, the most challenging is to know the extent of the membrane surface that is under the control of the clamp. Within any cell, the spread of current—and, hence, the voltage imposed by the clamp—is determined by the conductivity of the cell contents and its limiting membrane (Jack et al. 1983; Weiss 1996). These characteristics are encapsulated in the so-called space constant that defines how a current, injected at a point in space within a cell, dissipates with distance from that point. As the conductivity of the cytosol is generally three to four orders of magnitude higher than that of the cell membrane, the shape of the cell surface relative to the point of current injection becomes the most important determinant of current flow.

**Figure 3.**
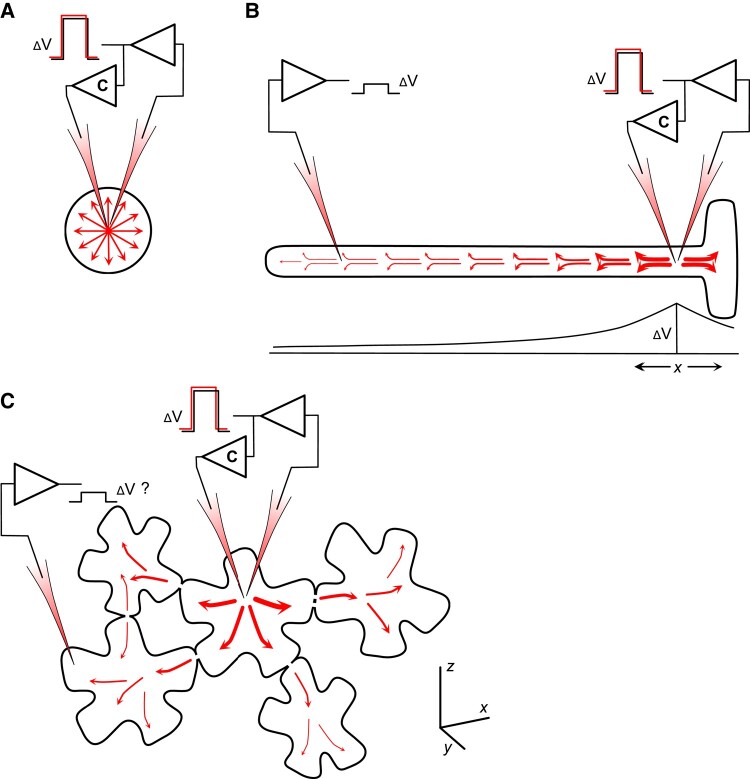
Current spread under voltage clamp in different cell geometries. Cell geometry is a critical factor in voltage clamp studies that make use of microelectrodes for clamp-current injection. Shown in each illustration is the current spread over the cell membrane as red arrows, with the arrow weight indicating the current density. In every case, the membrane is the primary resistance to ground (the external bath). The voltage clamp circuit in each illustration comprises two amplifiers (triangles). A voltage follower amplifier reports the membrane voltage from the right-most electrode; a comparator amplifier, labeled with C, compares this voltage with a command voltage input (not shown) and then returns a current to the cell through the other electrode(s) to correct for any difference between the recorded and command voltages. Also shown is a voltage step (ΔV) reported at the follower amplifier output, with the command step indicated in red and offset for visibility. **A)** Voltage clamp of a spherical or near-spherical cell ensures a uniform current distribution over the cell membrane. **B)** Voltage clamp of a near-cylindrical cell, here shown as a root hair cell, leads to current dissipation along the length of the cell as if it were an infinite cable. Thus, current passing across the membrane is reduced with distance away from the point of injection as current passes across the more proximal surfaces. The decline in the voltage step (ΔV) as the membrane current falls off with distance *x* is shown below. The follower amplifier recording voltage near the root hair tip sees only a fraction of this voltage change. **C)** Voltage clamp of a 3D matrix (*x, y, z* frame, *below*), comprising cells interconnected by plasmodesmata, leads to an ill-defined spread of current over the surfaces of the connected cells. Individual mesophyll cells may present a near-spherical shape, but their interconnections will draw down current locally. Most estimates (Spanswick 1972; Goldsmith and Goldsmith 1978) suggest that 10% to 20% of the current injected into a cell passes to the next through these connections. Thus, the challenge becomes one of defining the cellular interconnections and their spatial distributions.

For an accurate assessment of transport, the electrophysiologist needs current to flow equally over the membrane surface. Small cells with a near-spherical shape, for example protoplasts, are ideal for this purpose, as they ensure a uniform distribution of current over the membrane (Fig. 3A). Cells such as root hairs and the giant cells of the alga *Chara* behave like long conductive cables and are more difficult to work with, because current flow across the membrane will fall off with distance from the point of injection. So, the current driving a voltage change on the membrane will decline with distance, resulting in an indeterminant clamp voltage for the membrane as a whole. The space constant for Arabidopsis root hairs, for example, varies between 100 and 300 µm (Meharg et al. 1994). Thus, driving a current to clamp the membrane +100 mV from the free-running (unclamped) voltage at the base of a root hair is likely to achieve only a +30 mV displacement 200 µm away near the root hair tip (Fig. 3B). Of course, the consequence is that transporters at the base of the cell will see a very different membrane voltage from those near the tip, confounding any quantitative interpretation (Smith 1984).

These difficulties are further compounded by plasmodesmata that occur in the majority of multicellular plant tissues. Plasmodesmatal connections present a low-resistance pathway for current flow between cells that ensure electrical coupling with a spatial dissipation analogous to the current spread along an elongated cell (Spanswick 1972; Goldsmith and Goldsmith 1978). Plasmodesmata are usually distributed over the surfaces of cells without substantial orientational bias. So within the mesophyll of the leaf, for example, these connections create a 3D network (Fig. 3C) over which current spread is virtually impossible to define in practice (Eisenberg and Johnson 1970; Eisenberg et al. 1979; Jack et al. 1983). It is no surprise, then, that voltage clamp studies using intact plant tissues are confined almost entirely to single cells and to cells in tissues that are relatively easy to isolate from their neighbors.

One way around the problem of current spread in single, elongated cells is to use a linear, rather than a point, source for the current by inserting a fine wire through the length of the cell. This approach works well with the large and robust cells of *Chara* (Beilby and Coster 1979; Beilby 1986; Blatt et al. 1990a). For smaller and delicate cells such as root hairs, the alternative is to measure how the voltage change declines—and, hence, how current spreads—with distance from the point of injection and then correct for this behavior. Usually, the voltage decline can be approximated as a simple exponential function of distance. This approach works for changes imposed by a constant current and is suitable for studies of ion-coupled transport in the steady state (Meharg and Blatt 1995). It proves much more challenging to quantify current flow during transitions that are important for resolving relaxation characteristics of ion channel activation and deactivation. Such analyses require both temporal and spatial knowledge of the membrane voltage (Jack et al. 1983).

The difficulties of current spread are circumvented in intact guard cells, making work with these cells well-suited for the voltage clamp. This was the approach I championed from the start. It built on my experience with *Neurospora* and knowledge of mammalian physiology. Indeed, a long and distinguished history of research on mammalian as well as plant ion channels predates the patch clamp, only a very small part of which I touch on in this review. For those unfamiliar with this history (and for a few pedants who were), at first only the quantized transitions of single-channel recordings were recognized as *bona fide* evidence of channel activity (see Hedrich and Schroeder 1989). Several of these same laboratories now use the voltage clamp methods that I pioneered for guard cells. Of course, my laboratory also used the patch clamp to resolve the lifetime kinetics and regulation of several ion channels (Hamilton et al. 2000, 2001; Johansson et al. 2006; Lefoulon et al. 2018).

Guard cells are easy to isolate within epidermal peels and to mount and impale with microelectrodes. When mature, these cells lack functional plasmodesmata and are therefore isolated electrically from the neighboring epidermal cells (Wille and Lucas 1984; Erwee et al. 1985; Palevitz and Hepler 1985). Equally important, under the voltage clamp, current from an intracellular microelectrode spreads homogeneously over the membrane surface: analysis of the space constant, even for the large guard cells of *Vicia*, gave values near 300 to 500 µm (Blatt 1987a), more than an order of magnitude greater than the cell dimensions. In other words, voltage clamping these, and the much smaller guard cells of Arabidopsis, with a microelectrode—a point source—is clearly sufficient to ensure a uniform distribution of current over the cell membrane. If the questions were of transport and its regulation in the whole cell rather than of the conformational mechanics of a single channel, then the choice of the voltage clamp with intracellular microelectrodes was, for me, a “no brainer”. Why add the difficulties of preparing protoplasts, the uncertainties of their physiology, and the complications of cellular dialysis when measurements were possible from intact cells that were known to retain meaningful responses (Fischer 1968; Humble and Hsiao 1969; Lange et al. 1971; Willmer and Fricker 1996) to environmental and hormonal stimuli?

## The guard cell model

From the start, the need to establish the operational boundaries that constrain transport in the guard cell was abundantly clear: What voltages are typical of these cells? Which of the ions prevalent outside dominate the membrane conductance? Which ionic conductances respond to stimuli known to trigger stomatal movements? These are basic questions for which the answers are best derived from intact cells. An initial analysis (Blatt 1987b) showed that the plasma membrane of guard cells of *Vicia*, the model in common use at the time, was dominated by conductances to K^+^ and H^+^, was largely insensitive to Na^+^, and was only weakly dependent on Cl^−^ and Ca^2+^ outside. The sensitivity to H^+^ could be ascribed to H^+^-ATPases that, like the *Neurospora* (Sanders et al. 1981; Blatt and Slayman 1987) and *Chara* H^+^-ATPases (Kishimoto et al. 1984; Blatt et al. 1990a), transported one H^+^ per ATP hydrolyzed and introduced a significant conductance over much of the physiological voltage range (Blatt 1987a; Lohse and Hedrich 1992). Likewise, the K^+^ conductance was soon recognized to arise from two major K^+^ channel currents with opposing voltage sensitivities (Schroeder et al. 1987; Blatt 1988b; Schroeder 1988; Blatt and Clint 1989). These characteristics were supported by analyses with the H^+^-ATPase agonist fusicoccin and experiments with ^86^Rb^+^ as a radiotracer for K^+^ flux (Blatt 1988a; Blatt and Clint 1989; Clint and Blatt 1989). Of interest, the radiotracer experiments and supporting electrophysiological data (Clint and Blatt 1989) remain the only direct evidence for H^+^-coupled K^+^ uptake in guard cells and suggest that this pathway can account for as much as half of the cation uptake during stomatal opening.

Detailing the two K^+^ currents exposed a major divergence from the biophysical properties of K^+^ channels known in animals at the time. The outward-rectifying channels exhibited a dependence on K^+^ outside that interacted with the voltage action on the gate, what I perceived as a failsafe to ensure that the channels mediated K^+^ efflux for stomatal closure regardless of the K^+^ concentration outside (Blatt 1988b; Blatt and Gradmann 1997). This characteristic contrasted with the voltage dependence for gating of the inward-rectifying channels that was insensitive to K^+^ outside (Schroeder 1988; Blatt et al. 1990b; Blatt 1992). It was as if the animal membrane had been turned inside-out. Nerve, muscle, and invertebrate eggs incorporated inward-rectifying K^+^ channels with a K^+^ dependence that displaced channel gating with external K^+^ concentration (Armstrong and Binstock 1965; Hagiwara and Takahashi 1974) and was later attributed to block by intracellular ions (Lu and MacKinnon 1994; Taglialatela et al. 1994), especially Mg^2+^ (Vandenberg 1987; Agus and Morad 1991); by contrast, the animal outward-rectifying K^+^ channels—often identified as delayed rectifiers because of the association with the later, repolarization phase of the nerve and muscle action potential—showed little or no change in gating with external K^+^ (Hodgkin and Huxley 1952; Hodgkin et al. 1952; Wu and Haugland 1985), unlike the plant outward-rectifying K^+^ channels.

We now know that the inward- and outward-rectifying currents are carried principally by KAT1 and GORK, respectively, in Arabidopsis and their homologs in other species (Mueller-Roeber et al. 1995; Nakamura et al. 1995; Ache et al. 2000; Pilot et al. 2001; Hosy et al. 2003). Similar characteristics show up in the few grass stomata that have been characterized (Fairley-Grenot and Assmann 1992; Buchsenschutz et al. 2005). Furthermore, we have a good understanding of the mechanics of their gating with voltage and its dependence on the conformation of the so-called voltage sensor domain (Hoshi 1995; Blatt and Gradmann 1997; Marten and Hoshi 1997; Zei and Aldrich 1998; Eisenach et al. 2014; Lefoulon et al. 2018; Lefoulon 2021). This understanding is supported by the crystal structures (Table 1) for KAT1 (Clark et al. 2020), the related AKT1 channel (Dickinson et al. 2022; Lu et al. 2022) and the SKOR channel (Li et al. 2023) that is near sequence-identical with GORK. These channels are all members of the cyclic-nucleotide binding domain (CNBD) channel subfamily (Jegla et al. 2018) that includes the cardiac pacemaker HCN channels (Moroni et al. 2001; Lee and MacKinnon 2017). How gating in GORK and its homologs is coupled with extracellular K^+^ remains a matter of interest, not least because it is likely to underpin the rapid stomatal movements of many plants with C_4_ photosynthesis (Alvim 2022) and has implications for the channel protein dynamics (Eisenach et al. 2014). My laboratory recently found that manipulating this coupling yields substantial gains in both water use efficiency and photosynthetic carbon assimilation when light fluctuates, as it commonly does in the environment (Blatt and Alvim 2022; Horaruang et al. 2022).

**Table 1.** Ion channel and aquaporin structures and gating relevant to guard cells^a^

Channel	Gene	PDB code	Gating factors	Phosphorylated by	Function	Refs
**K^+^ channels**						
KAT1	At5g46240	6V1Y	V, [Ca^2+^]_i_, [H^+^]_o,_ [H^+^]_i_	CPK3,CPK6,OST1	K^+^ uptake	^b^
AKT1	At2g26650	7T4X,7WSW, 7FCV, 7WM2	V	CBL1/CIPK23	K^+^ uptake	^c^
SKOR	At3g02850	8JET, 8JEU	V, [H^+^]_o,_ [H^+^]_i_		K^+^ efflux	^d^
TPC1	At4g03560	5E1J, 7HFK, 5DQQ	V, [Ca^2+^]_o_, [H^+^]_o,_ [H^+^]_i_		Unknown	^e^
**Anion channels**						
SLAC1	At1g12480	7EN0, 7WNQ	V, [Ca^2+^]_i_, [H^+^]_i_, [Ac]_i_	HT1,OST1,CBL1/CIPK6, CPK3, CPK6	Cl^−^/NO_3_^−^ efflux	^f^
ALMT12	At4g17970	7W6K	V, [Ca^2+^]_i_, [H^+^]_i_, [Mal]_o_	OST1,CBL1/CIPK6	Cl^−^/Mal efflux	^g^
**Aquaporins**						
PIP2;1	At3g53420	1Z98, 3CN5, 4JC6	[Ca^2+^]_i_, [H^+^]_o_, [H^+^]_o_	OST1, SnRK2.8	H_2_O flux	^h^
TIP2;1	At3g16240	5I32	[H^+^]_i_		H_2_O flux	^i^

^a^Tabular summary of gene and structural detail available to date for ion and aquaporin channels pertinent to guard cells along with a brief summary of gating factors and protein kinases known to act on channel activity. See Table IX of Jezek and Blatt (2017) for an extensive summary of kinases and phosphatases targeting the channels.

^b^ Clark et al. (2020).

^c^ Dickinson et al. (2022) and Lu et al. (2022).

^d^ Li et al. (2023).

^e^ Guo et al. (2016) and Kintzer and Stroud (2016).

^f^ Chen et al. (2010a, 2010b), Deng et al. (2021), and Li et al. (2022).

^g^ Qin et al. (2022).

^h^ Tornroth-Horsefield et al. (2006).

^i^ Kirscht et al. (2016).

Early evidence for the function of anion channels in guard cells surfaced as a background conductance that was enhanced 2-fold within minutes of exposing the cells to abscisic acid, which normally triggers stomatal closure (Blatt 1990; Thiel et al. 1992; Grabov et al. 1997), and later to CO_2_ (Brearley et al. 1997). The quasi-linear characteristic of this conductance was consistent with a Ca^2+^-sensitive leak observed in guard cell protoplasts (Schroeder and Hagiwara 1989) but was at odds with the sharp voltage dependence of what was subsequently labeled as GCAC1 (Guard Cell Anion Channel 1), the “rapid activating” or R-type anion channel (Keller et al. 1989; Hedrich et al. 1990; Marten et al. 1992). A definitive study by Schroeder and Keller (1992) clarified the relationship between the two currents, the first a slow, or S-type, anion current that activated and deactivated over 20 to 30 s and the second the rapid, R-type, current that responded to voltage with halftimes of 50 to 100 ms.

The S-type current is now recognized to arise with the SLAC1 channel and its homolog SLAH3 (Negi et al. 2008; Vahisalu et al. 2008; Geiger et al. 2011), members of the structurally similar dicarboxylate transporter family (Chen et al. 2010a). The R-type current was associated with ALMT12 (Meyer et al. 2010), a member of the aluminium-sensitive malate transporter family of proteins (Table 1). However, the Arabidopsis *almt12* mutant retains the R-type current (Sasaki et al. 2010), and even double and triple *almt* mutants show a near-wild-type current in vivo (Jaslan et al. 2023). These observations close the door on assertions that the R-type anion current is mediated by *ALMT* gene products and leave its identity unresolved.

Molecular identities aside, the distinctive contributions of the S- and R-type anion currents to stomatal closure was a matter of controversy for some time. Their respective contributions were subsequently resolved through a computational analysis incorporating the quantitative characteristics of guard cell transport (Chen et al. 2012; Minguet-Parramona et al. 2016). The analysis shows that while the S-type current favors an overall bias to more positive voltages, the R-type current is instrumental in the repetitive membrane depolarizations, the action potentials, that arise through coupling with K^+^ and Ca^2+^ channels and [Ca^2+^]_i_ elevations to accelerate a rapid loss of K^+^, Cl^−^, and malate from guard cells (Blatt and Thiel 1994; Grabov and Blatt 1998; Allen et al. 1999; Hamilton et al. 2000; Chen et al. 2010b; Stange et al. 2010). [It is difficult to reconcile the claim that the *almt12* mutant slows stomatal closure in high CO_2_ (Jaslan et al. 2023), given the wild-type characteristics of the current and an analysis of the R-type current in guard cell action potentials and stomatal closure (Chen et al. 2012; Minguet-Parramona et al. 2016); instead, the question arises whether the *almt12* mutant harbors additional T-DNA insertions.]

## Guard cell Ca^2+^ channels and [Ca^2+^]_i_ oscillations

**Figure 4.**
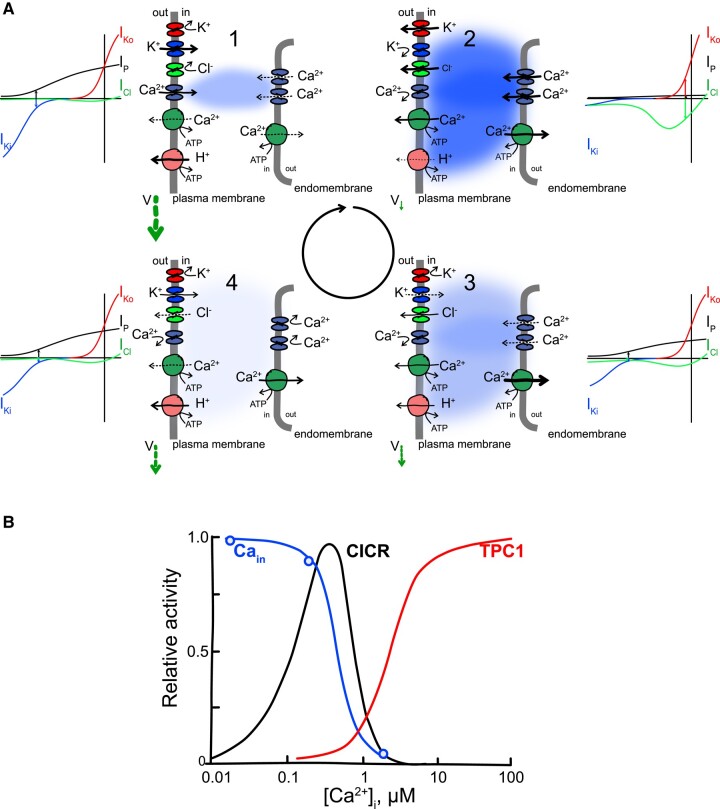
Ca^2+^-dependent gating of Ca^2+^ channels needed to support Ca^2+^-induced Ca^2+^ release. **A)** The cycle of Ca^2+^-induced Ca^2+^ release and its recovery progresses clockwise through four steps in guard cells (see Blatt 2000). Outer insets at each step illustrate the component current-voltage curves for the plasma membrane H^+^-ATPase (I_P_, *black*), the two K^+^ channels (I_Ki_, *blue*; I_Ko_, *red*), and the Cl^−^ channel (I_Cl_, *green*). The free-running membrane voltage in each is indicated by the position of the opposing arrows along the voltage axis (color-coded to the dominant current in each case) and below each cartoon frame (V, *dark green arrows*). (1) Ca^2+^ influx across the plasma membrane, driven by the H^+^-ATPase and membrane hyperpolarisation, triggers endomembrane Ca^2+^-permeable channels to activate. (2) Endomembrane Ca^2+^ release floods the cytosol to raise [Ca^2+^]_i_ to micromolar concentrations, suppressing inward-rectifying K^+^ channel and H^+^-ATPase activities and promoting Cl^−^ channel activity, thereby depolarizing the plasma membrane. (3) [Ca^2+^]_i_ elevation and membrane depolarization promote Ca^2+^-ATPases to resequester the cation and remove it to the apoplast. (4) The lowered [Ca^2+^]_i_ reduces Cl^−^ channel activity, promotes inward-rectifying K^+^ channel activity and allows the H^+^-ATPase to re-activate, thereby repolarising the plasma membrane. The outward-rectifying K^+^ channel activity is insensitive to [Ca^2+^]_i_ and parallels that of the Cl^−^ channels, driven principally by the changes in membrane voltage [see also Chen et al (2012) and Blatt (2000)]. **B)** Ca^2+^-dependent gating characteristics of the 13 pS, plasma membrane Ca^2+^ channel (Ca_in_) is consistent with Ca^2+^-dependent inhibition expected of a channel that overlaps with, and triggers, Ca^2+^-induced Ca^2+^ release [data redrawn from Hamilton et al (2000, 2001)]. The Ca^2+^-dependent gating characteristics of the dominant Ca^2+^ release pathway (CICR) requires both Ca^2+^-dependent activation at submicromolar [Ca^2+^]_i_, overlapping with the activity of the triggering Ca^2+^ channels, and Ca^2+^-dependent inactivation at supramicromolar [Ca^2+^]_i_ [curve redrawn from Bezprozvanny et al (1991)]. These characteristics are not a feature of TPC1, which shows only Ca^2+^-dependent activation at [Ca^2+^]_i_ beyond the effective range for CICR [curve redrawn from data of Schulz-Lessdorf et al (1995) and Pei et al (1999)].

Remarkably, although their physiological characteristics are well-established, the molecular identities of the Ca^2+^ channels that mediate [Ca^2+^]_i_ increases remain one of the major unsolved mysteries of the guard cell. These channels play a central role in triggering the [Ca^2+^]_i_ transients that coordinate transport for stomatal closure (Grabov and Blatt 1998; Blatt 2000; Hamilton et al. 2000; Pei et al. 2000; Allen et al. 2001; Koehler and Blatt 2002; Roelfsema and Hedrich 2010; Jezek and Blatt 2017). It was a chance observation that led Alex Grabov and me (Grabov and Blatt 1998) to connect [Ca^2+^]_i_ transients in guard cells with membrane voltage. These studies, combining the voltage clamp with the Ca^2+^-sensitive dye Fura2, showed a steep rise in [Ca^2+^]_i_ with membrane hyperpolarization. From these findings, we identified the dominant, voltage-activated Ca^2+^ channel in the guard cell plasma membrane, its activation by ABA (Hamilton et al. 2000, 2001), and its role in triggering endomembrane Ca^2+^ release (Grabov and Blatt 1999; Garcia-Mata et al. 2003). Together with our concurrent work (Grabov and Blatt 1997, 1999) quantifying the kinetics of the inward-rectifying K^+^ channels and their dependence on [Ca^2+^]_i_, it was soon clear (Fig. 4A) that the voltage and [Ca^2+^]_i_ oscillations were coupled within a cycle of K^+^ and Cl^−^ efflux to accelerate solute loss (Blatt 2000). Later studies quantifying the [Ca^2+^]_i_-dependence of the guard cell R- and S-type anion channels (Mori et al. 2006; Chen et al. 2010b; Meyer et al. 2010; Stange et al. 2010) only added depth to this conclusion. A central lesson here was that the [Ca^2+^]_i_ oscillations “reflect a spectrum of frequencies that emerge from the balance of intrinsic transport activities” in the guard cell and the interactions between Ca^2+^, K^+^, and Cl^−^ flux (Minguet-Parramona et al. 2016) rather than a unique [Ca^2+^]_i_ frequency that “encodes” stomatal closure.

What do we know about the plasma membrane Ca^2+^ channels? Hamilton et al. (2000, 2001) reported a Ca^2+^-permeable channel with a limiting single-channel conductance of 13 pS, like many Ca^2+^ channels an equal or greater permeability to Ba^2+^, and a high selectivity (>10:1) for the divalents over K^+^ and Cl^−^. The channel showed a significant voltage dependence with an apparent gating charge of unity, possibly higher (Koehler and Blatt 2002; Sokolovski et al. 2008), thus distinguishing the Ca^2+^ channel from K^+^ channels, including KAT1 and GORK, that show gating charges of 1.8 to 2.2. [In whole-cell analysis, channel gating can be described with two parameters: the gating charge defines the slope of the change in relative activity (relative open probability) with voltage; the midpoint voltage defines the voltage at which this activity is half-maximal. While the channel midpoint voltage is often under ligand or environmental control, the gating charge is a unique “fingerprint” intrinsic to the structure of the channel protein.] The mean open probability of the Ca^2+^ channel was enhanced over 100-fold within 10 to 20 s of adding ABA to excised, inside-out membrane patches, leading to a +40 mV shift in the midpoint voltage for the relative open probability; an equivalent, but slower, response in cell-attached recordings implied that that ABA must traverse the membrane before binding to its intracellular receptor (Hamilton et al. 2000).

Concurrent work by Pei et al. (2000) similarly uncovered a divalent cation-selective current that was enhanced by reactive oxygen species and abscisic acid treatment, but these studies provided little other quantitative detail. Hamilton et al. (2000, 2001), Koehler and Blatt (2002), and Koehler et al (2003) reported channel mean open probabilities around 0.0003 to 0.001 near −120 mV that rose rapidly at more negative voltages and channel activation by reactive oxygen species with a K_1/2_ of 76 µM. Unusually, the channel open probability was strongly enhanced, with a Hill (binding) coefficient near 4, as the external divalent concentration rose above 2 mM; this effect resulted primarily from an increase in the open lifetime of the channel. These studies also showed that phosphorylation of the Ca^2+^ channels was a prerequisite for their subsequent response to ABA. Finally, the channel activity was strongly dependent on internal Ca^2+^, falling roughly 10-fold as [Ca^2+^]_i_ increased from 200 nM to 2 µM (Hamilton et al. 2000). Suppression by [Ca^2+^]_i_ was precisely the feedback necessary to support [Ca^2+^]_i_ transients and their temporal oscillatory behavior, indicating that the 13 pS channel was responsible for triggering the [Ca^2+^]_i_ oscillations observed in vivo.

Could these Ca^2+^ channels be ascribed to one or more assemblies of cyclic nucleotide-gated channel (CNGC) subunits? Possibly, but the evidence to date is not convincing (Table 2). Cyclic nucleotide-gated channels incorporate a structure, broadly similar to plant CNBD K^+^ channels, that comprises six transmembrane-spanning domains (Kohler et al. 1999) with sites for calmodulin binding (Kohler and Neuhaus 2000; DeFalco et al. 2016). Certainly, calmodulin binding and its regulation of channel activity might explain the negative feedback evident with [Ca^2+^]_i_ elevation (Fischer et al. 2017; Pan et al. 2019; Jarratt-Barnham et al. 2021). However, other characteristics exhibited by CNGCs to date do not match those of the plasma membrane Ca^2+^ channels first described by Hamilton et al (2000, 2001). With the possible exception of CNGC18 (Gao et al. 2014), CNGC channels -including the CNGCs of guard cells (Wang et al. 2013b)—are broadly permeant to monovalent as well as divalent cations (Dietrich et al. 2020; Jarratt-Barnham et al. 2021). Furthermore, CNGCs uniformly show shallow voltage sensitivities with gating charges of 0.2 to 0.4, even in the presence of cytosolic Mg^2+^ (Lemtiri-Chlieh et al. 2020). Tan et al. (2023) reported that a quadruple knockout of the four dominant CNGCs in Arabidopsis guard cells slows the [Ca^2+^]_i_ oscillations evoked by ABA. The oscillations are still present, however. Furthermore, reactive oxygen species elicit [Ca^2+^]_i_ elevations in the quadruple knockout mutant, much as they do in the wild type. So, yes, CNGCs may contribute to [Ca^2+^]_i_ homeostasis in guard cells, but the molecular identity of the 13 pS Ca^2+^ channel most likely lies somewhere else.

**Table 2.** Comparison of guard cell Ca^2+^ channels, candidate channel proteins and their gating^a^

Channel	Gene	Voltage dependence		Action: [Ca^2+^]_o_	[Ca^2+^]_i_	Other	Refs
		δ	V_1/2_ (mV)				
**Plasma membrane**							
Ca_in_	Unknown	1 to 1.4	−140 to −80	+shift V_1/2_	K_inh_, 350 nM, *h* = 4	ROS act	^c^
					− shift V_1/2_	ABA act	
CNGC5	*CNGC5*	<0.4^b^	<−160^b^	n.d.	CAM-mediated K_inh_	ROS insens	^d^
CNGC6	*CNGC6*	<0.4^b^	<−160^b^	n.d.	CAM-mediated K_inh_	ROS insens	^d^
CNGC9	*CNGC9*	<0.4^b^	<−160^b^	n.d.	CAM-mediated K_inh_	ROS insens	^d^
CNGC12	*CNGC12*	<0.4^b^	<−160^b^	n.d.	CAM-mediated K_inh_	ROS insens	^d^
**Tonoplast and endomembranes**							
CICR	Unknown	3 to 4	−80 to −40	+shift V_1/2_	K_act_, 400 nM, *h* = 4	−shift V_1/2_, HCO_3_^−^	^e^
					K_inh_, 1.2 µM, *h* = 4		
SV	*TPC1*	1 to 1.4	+40 to +90	+shift V_1/2_	K_act_, 3 µM, *h* = 4	inhib, pH < 7	^f^
					no K_inh_	[Mg^2+^]_i_ act	
IP_3_R	Unknown	∼1^b^	−80 to −50^b^	n.d.	no direct K_act_	IP_3_ > 1 µM, act	^g^
					no direct K_inh_		
IP_6_R	Unknown	n.d.	n.d.	n.d.	n.d.	IP_6_ > 1 µM, act	^h^
cADPR	Unknown	<1^b^	−120 to −80^b^	n.d.	No direct K_act_	cADP > 20 nM act	^j^
					K_inh_, 500 nM^b^		

^a^act, activation; inh, inhibition; insens, insensitive; K_act_, activation K_1/2_; K_inh_, inhibition K_1/2_; *h*, Hill coefficient; V_1/2_, conductance midpoint voltage; ±shift, positive/negative shift in V_1/2_; n.d., not determined.

^b^Estimated from published data.

^c^ Grabov and Blatt (1998), Hamilton et al. (2000, 2001), Pei et al. (2000), Koehler and Blatt (2002), Garcia-Mata et al. (2003), Sokolovski et al. (2005, 2008), and Wang et al. (2013a).

^d^ Wang et al. (2013b), Pan et al. (2019), Dietrich et al. (2020), Lemtiri-Chlieh et al. (2020), and Tan et al. (2023).

^e^Minimal OnGuard parameters established for endomembrane Ca^2+^ release (Chen et al. 2012; Hills et al. 2012; Wang et al. 2017; Jezek et al. 2021; Blatt et al. 2022).

^f^ Hedrich and Neher (1987), Ward and Schroeder (1994), Schulz-Lessdorf and Hedrich (1995), Pei et al. (1999), Pottosin et al. (2004), Beyhl et al. (2009), and Hedrich and Marten (2011).

^g^ Alexandre et al. (1990), Blatt et al. (1990a, 1990b), Gilroy et al. (1990), and Allen et al. (1995).

^h^ Lemtiri-Chlieh et al. (2000, 2003).

^j^ Allen et al. (1995), Muir et al. (1997), and Leckie et al. (1998).

## Probing the tonoplast

The tonoplast membrane surrounding the guard cell vacuole presents many more challenges. As the vacuole comprises some 80% to 90% of the volume of the guard cell, the major fraction of solute and water flux occurring during stomatal movements must cross both membranes (Willmer and Fricker 1996; Jezek and Blatt 2017). It is no surprise, then, that transport across the tonoplast is coordinated with that of the plasma membrane. Both membranes share a common pool of solutes within the cytosol, and the transport of these solutes, including that of Ca^2+^, are affected by, and impact on, the activities at both membranes (Horaruang et al. 2020).

There are substantial technical challenges in gaining access to transport across the tonoplast. Analysis in vivo is limited to radiotracer methods (MacRobbie 1995, 2000; MacRobbie and Kurup 2007) that, with few exceptions (Clint and Blatt 1989), yield data only for the sum fluxes of any one ion. Patch clamp methods, by contrast, have provided much quantitative data on the individual channels and pumps activities from isolated vacuoles. These include the biophysical and regulatory characteristics of the slow- (SV) and fast-activating (FV) and the voltage-independent (VK) K^+^ permeable channels (Hedrich and Neher 1987; Weiser and Bentrup 1991; Ward and Schroeder 1994; Schulz-Lessdorf and Hedrich 1995; Allen et al. 1998), the SV and VK channels now recognized as the two-pore channel proteins TPC1 and TPK1, respectively (Furuichi et al. 2001; Bihler et al. 2005; Peiter et al. 2005; Gobert et al. 2007). Like work with membrane vesicles, however, patch recordings of the tonoplast require some educated guesswork to define the environment on the cytosolic side of the membrane. As a result, there remain many more mysteries that have yet to be resolved about the components, nature and regulation of tonoplast transport as it operates in vivo.

The growing interest in transport across other endomembranes, especially the endoplasmic reticulum and nuclear envelope, chloroplasts, and mitochondria (De Angeli et al. 2009; Chanroj et al. 2011; Furumoto et al. 2011; Loro and Costa 2013; Mueller et al. 2014; Maurel et al. 2015; Charpentier et al. 2016; De Col et al. 2017; Leitão et al. 2019), faces very similar challenges. Among others, these compartments contribute to the [Ca^2+^]_i_ characteristics of plant cells and include the endoplasmic reticulum (Garcia-Mata et al. 2003; Blatt et al. 2007; Bonza et al. 2013), the vacuole (Allen et al. 1995; Beyhl et al. 2009) and, over a higher [Ca^2+^]_i_ range, also mitochondria and chloroplasts (McAinsh and Pittman 2009; Loro et al. 2012; Loro and Costa 2013). There is not space here to cover these topics, but they are bound to add substantially to an understanding of cellular dynamics and signaling (Resentini et al. 2021).

Among the defining mysteries, the pathways of endomembrane Ca^2+^ release for [Ca^2+^]_i_ transients and oscillations remain to be identified. This is a challenge that, by historical circumstance, is tied hand-in-hand with the puzzle that is the TPC1 (SV) channel. The SV current was originally identified as a Ca^2+^-activated and cation-permeable channel of 60 pS that rectified strongly into the beet vacuole (Hedrich et al. 1988), by convention hereafter defined as an outward-rectifying current (Bertl et al. 1992). The current was later identified with a tandem-pore channel (TPC) analogous to the mammalian channels carrying the same moniker (Furuichi et al. 2001; Peiter et al. 2005) and has since proven to be a dominant feature of the vacuoles of the cells in virtually all land plants (Hedrich and Marten 2011). TPC1 current is activated by [Ca^2+^]_i_ elevation on the cytosolic side of the membrane (Hedrich and Neher 1987) that promotes a permeability also to Ca^2+^ (Ward and Schroeder 1994; Schulz-Lessdorf and Hedrich 1995), but the current is much reduced at the acid pH values typical of the vacuole (Schulz-Lessdorf and Hedrich 1995; Beyhl et al. 2009). Thus, the TPC1 current is recovered at voltages negative of 0 mV only with supramicromolar [Ca^2+^]_i_ concentrations (Pottosin and Schonknecht 2007; Hedrich and Marten 2011). As tonoplast voltages normally range from −20 to −70 mV (Spanswick and Williams 1964; Bentrup et al. 1986), the first, and foremost puzzle is simply why the current activates only well outside the physiological voltage range.

It has proven equally difficult to “shoehorn” the [Ca^2+^]_i_ dependence of TPC1 into the features essential for evoked Ca^2+^ release (Fig. 4B), the phenomenon of Ca^2+^-induced Ca^2+^ release (CICR) that underpins guard cell [Ca^2+^]_i_ transients (Grabov and Blatt 1999; Blatt 2000; Garcia-Mata et al. 2003; Chen et al. 2012; Minguet-Parramona et al. 2016). Yet, there is unequivocal evidence that the vacuole is a major endomembrane Ca^2+^ store (MacRobbie 1992; Allen and Sanders 1994; Pottosin and Schonknecht 2007; Pittman 2011) and that it contributes to [Ca^2+^]_i_ transients in vivo in guard cells in response to changes in light and CO_2_ (Jezek et al. 2021). As Mareike Jezek and I noted before (Jezek and Blatt 2017), one of the prerequisites for [Ca^2+^]_i_ transients and oscillations is that the pathways for Ca^2+^ entry to the cytosol must be self-limiting; for endomembrane Ca^2+^ release, this requirement implies a bell-shaped dependence on [Ca^2+^]_i_ so that the Ca^2+^ flux shuts down at supramicromolar [Ca^2+^]_i_ to avoid catastrophic Ca^2+^ release.

The activity of TPC1 only rises with [Ca^2+^]_i_ and with an apparent K_1/2_ near 3 µM and Hill coefficient of 4 (Hedrich and Neher 1987; Ward and Schroeder 1994; Schulz-Lessdorf and Hedrich 1995), values that also place TPC1 activation outside the range needed for triggered [Ca^2+^]_i_ increases (Fig. 4B). [Note that Pei et al. (1999) erroneously quote a K_1/2_ of 227 nM for Ca^2+^ activation of the SV (TPC1) current, even though their data clearly shows a value near 3 µM, an order of magnitude greater (Hills et al. 2012).] Thus, it is difficult to see how the activation of TPC1 might contribute to anything other than cellular collapse. What, then, is the role of TPC1? To date, in vivo studies with the Arabidopsis *tpc1* null mutant and the *fatty acid oxygenation upregulated2* (*fou2*) mutant, which renders TPC1 largely insensitive to vacuolar Ca^2+^, have failed to uncover a phenotype attributable to [Ca^2+^]_i_ signaling (Peiter et al. 2005; Islam et al. 2010; Hedrich and Marten 2011). Thus, a question mark hangs over the function of TPC1, as it has for over 35 years.

All estimates indicate that 95% of the Ca^2+^ flux for [Ca^2+^]_i_ transients comes from endomembrane stores (Grabov and Blatt 1999; Chen et al. 2012; Jezek et al. 2021), much as in animals (Bezprozvanny et al. 1991; Hille 2001). So, which channels, then, might facilitate CICR and [Ca^2+^]_i_ oscillations in guard cells? Several Ca^2+^-permeable channels (Table 2) associated with endomembrane stores are activated by submicromolar [Ca^2+^]_i_ and by ligands, including IP_3_, cyclic ADP-ribose (cADPR), nitric oxide (NO), and inositol hexakisphosphate (IP_6_), and have been implicated in Ca^2+^ release (Alexandre and Lassalles 1990; Blatt et al. 1990b; Muir and Sanders 1996; Wu et al. 1997; Leckie et al. 1998; Grabov and Blatt 1999; Garcia-Mata et al. 2003; Lemtiri-Chlieh et al. 2003). Furthermore, there is unequivocal evidence for ryanodine- and IP_3_-sensitive Ca^2+^ release behind CICR in guard cells (Blatt et al. 1990b; Gilroy et al. 1990; Grabov and Blatt 1999). However, no Ca^2+^-permeable channel has yet to be resolved at the tonoplast or at any other endomembrane with the bell-shaped dependence on [Ca^2+^]_i_ required for CICR (Bezprozvanny et al. 1991; Hille 2001). I have no doubt that solving this mystery will open entirely new perspectives on signaling in plants that extend far beyond the guard cell.

## Modeling guard cells

At a major conference in 2004, a delegate asked of me “Why do you work on guard cells when they’re so complex?” The delegate, an internationally renowned colleague, had worked for decades on wounding and plant pathogen defence. So, the irony of the situation was not lost on me. [Another colleague once quipped that plant pathogenesis is the study of multiply nested double-negatives—as a linguistic construct, a usage that can be difficult to parse even when not nested (Pinker 2014)]. At the start of this article, I noted that by the turn of this century, the sheer volume of data for transport, especially in guard cells, was overwhelming. I have said very little of signaling and transport regulation, beyond mentioning [Ca^2+^]_i_ and reactive oxygen species, and nothing of the regulatory impacts of cytosolic pH, nitrosylation, persulfidation, or phosphorylation, let alone of gene expression and vesicle trafficking for transport protein delivery to, and recycling from, target membranes.

Of course, signaling processes are intimately connected with our understanding transport and add to this body of data [cf. Kollist et al. (2014), Rodriguez and Shan (2022), Hedrich and Geiger (2017), Lefoulon (2021), Karnik et al. (2017), Chen et al. (2016), Papanatsiou et al. (2015), Pantaleno et al. (2021), and Jezek and Blatt (2017)]. They offer important insights into how guard cells coordinate transport for stomatal movements. Yes, their impacts are multifaceted, but these can be tiered with clear patterns to the physiology. For example, Lefoulon (2021) pointed out that phosphorylation of K^+^ channels, almost without exception, affects ensemble current amplitude rather than gating. In other words, phosphorylation alters the pool size of channels available for activation by voltage but does not alter their sensitivity to voltage. The same characteristics also to apply to Ca^2+^ and Cl^−^ channels (Koehler and Blatt 2002; Assmann and Jegla 2016; Eisenach et al. 2017; Jezek and Blatt 2017; Li et al. 2022), and H^+^-coupled Cl^−^ transport is similarly affected by phosphorylation in amplitude only (Wege et al. 2014).

My colleague's comment was valid in one sense, though perhaps not as it was intended: The kinetics of most biological processes are highly non-linear, often with respect to multiple variables, for ion transport including voltage. Interactions between these processes, not least through the entanglements of a common membrane voltage and the pool of cytosolic solutes shared between the plasma membrane and tonoplast (see Current matters, above). These characteristics give rise to physiology that is frequently unexpected or counterintuitive, what is termed “emergent” behavior. Simply put, by the turn of this century, it was obvious that intuition was a very poor guide to understanding how stomata work.

While in Cambridge, I often stopped by the Physiological Laboratories on the Downing site adjacent Plant Sciences to share a coffee with V.L. (Arieh) Lew and discuss the challenges of transport interactions. We continued these discussions long after I left Cambridge for my first substantive posting at Wye College in Kent in 1990. Arieh had developed mechanism-based, mathematical models of the erythrocyte (Lew and Bookchin 1986) that explained much of the phenomenology of Gardos dehydration (Gardos 1958) and of sickle-cell anemia with counterintuitive predictions that were subsequently validated in experiments (Lew and Bookchin 2005; Lew 2022). It was obvious to us the need to approach the problems of stomatal physiology—for example, how guard cells respond to abscisic acid—using similar, mechanism-based strategies. Eventually, we secured funding from the UK Biotechnology and Biological Sciences Research Council and embarked on this project in 2008.

[As an aside, Wye College, part of the University of London, gained an international reputation in large measure from the work of agricultural chemist Ralph Louis Wain CBE, FRS. During the 1950s, 1960s and 1970s while at Wye College, Wain developed, synthesized, and studied the actions of a number of auxin-like herbicides and was instrumental in discovering the role of ABA in regulating foliar transpiration (Cornforth et al. 1966; Wright and Hiron 1969; Hiron and Wright 1973). By chance, my own laboratory while at the College focused on ABA, work that led to our discoveries, noted earlier, of the guard cell Ca^2+^ channel and of voltage and [Ca^2+^]_i_ coupling in K^+^ and Cl^−^ flux control (Grabov and Blatt 1997, 1998, 1999; Blatt 2000; Hamilton et al. 2000; Koehler and Blatt 2002; Sokolovski et al. 2008). A second discovery of ABA action through vesicle trafficking (SNARE) proteins (Leyman et al. 1999) led my laboratory at Glasgow to SNARE-K^+^ channel binding and their coregulation in cell expansion (Honsbein et al. 2009; Grefen et al. 2010, 2015; Karnik et al. 2017; Lefoulon et al. 2018; Waghmare et al. 2019). Wye College was subsumed within Imperial College London in 2000, shortly before I left to take up the Regius Chair of Botany at the University of Glasgow. The merger was poorly handled and its oversight unsympathetic to Wye and its surrounds. Wye College closed in 2009.]

Stomata are ideally suited to the “bottom-up” approach of mechanistic, computational modeling. Their movements are driven by changes in the osmotic solute contents of the guard cells that are connected to cell volume, turgor, and stomatal aperture. The osmotic solute contents, in turn, are governed by ion and water flux that connect to the relevant ion gradients and permeabilities across each membrane. Finally, the operation of each of the 23 major solutes and water transporters can be described quantitatively within sets of well-established kinetic equations, fully constrained by experimental data and by physical laws of mass and charge conservation (Table 3). Even though our knowledge of transport at the tonoplast was less well-developed, there was sufficient detailed information from experiments (Blatt 2000; Webb et al. 2001; Hetherington and Brownlee 2004; Blatt et al. 2007; McAinsh and Pittman 2009; Assmann and Jegla 2016; Jezek and Blatt 2017) to constrain the range of parameters needed to comply with experimental results (Chen et al. 2012).

**Table 3.** A minimal set of solute transporters used by the OnGuard platform^a^

Transporter^b^	Representative gene product^c^	Transported ions	Gating^e^: [Ca^2+^]_i_	[H^+^]_i_	Function^f^
**Plasma membrane**					
H^+^-ATPase	AHA1, AHA2	H^+^	inhib	substr	Energisation
Ca^2+^-ATPase	ACA8, ACA10	Ca^2+^, H^+^	substr		Ca^2+^ efflux
H^+^–K^+^ symport	HAK5	K^+^, H^+^		substr	K^+^ influx, high affinity
H^+^-anion symport	NPF7	Cl^−^(NO_3_^−^), H^+^		substr	Cl^−^ influx, high affinity
H^+^-Mal symport	ABCB14	Mal, H^+^		substr	Mal influx
Ca^2+^ channel (in)^d^	(unknown)	Ca^2+^	inhib		Ca^2+^ influx, CICR
K^+^ channel (in)	KAT1, KAT2	K^+^	inhib	activ	K^+^ influx
K^+^ channel (out)	GORK	K^+^		inhib	K^+^ efflux
K^+^ channel (none)	(unknown)	K^+^			K^+^ leak
Anion channel (weak)	SLAC1, SLAH3	Cl^−^(NO_3_^−^)	activ	activ	Anion efflux
Anion channel (out)	(unknown)	Cl^−^, Mal	activ	activ	Anion efflux
Aquaporin	PIP2;1		inhib		Water flux
**Tonoplast**					
H^+^-ATPase	VHA1	H^+^		substr	Energisation
H^+^-PPase	PVA1	H^+^		substr	Energisation
Ca^2+^-ATPase	ACA4, ACA11	Ca^2+^, H^+^	substr		Ca^2+^ efflux
Cl^−^(NO_3_^−^) channel	ALMT9	Cl^−^(NO_3_^−^)			Anion influx
Mal channel	ALMT6	Mal			Mal influx
H^+^/Ca^2+^ antiport	CAX2, CAX3	Ca^2+^, H^+^	substr	substr	Ca^2+^ efflux
H^+^/Cl^−^ antiport	CLC1	Cl^−^(NO_3_^−^), H^+^		substr	Cl^−^(NO_3_^−^) efflux
Ca^2+^ channel (in)^d^	(unknown)	Ca^2+^	inhib	inhib	Ca^2+^ influx
K^+^ channel (weak)	TPK1	K^+^	activ	activ	K^+^ influx
K^+^ channel (none)	FV	K^+^	inhib	inhib	K^+^ leak
K^+^/Ca^2+^ channel (out)^g^	TPC1	K^+^, Ca^2+^	activ	inhib	(unknown)
Aquaporin	TIP1;1, TIP2;1		inhib		Water flux

^a^Listed transporters are essential for osmotically-active solute, H^+^ and Ca^2+^ flux. See Chen et al (2012), Jezek and Blatt (2017), and Jezek et al. (2021) for a complete list of the transporter properties.

^b^Transporters groups by functional characteristics. Channel rectification and charge flux direction is indicated in parentheses.

^c^Representative Arabidopsis gene products, where known.

^d^Channels identified by physiological characteristics, yet to be associated with a gene product.

^e^activ, activation; inhib, inhibition; substr, transported solute (substrate or product).

^f^Ion (chemical) flux direction relative to the cytosol.

^g^Function of TPC1 remains unknown and is included here as a placeholder only.

Ultimately, for the guard cell, the important outputs are the cell volume and osmolality, water potential and turgor, the voltages across each membrane, the predominant ion concentrations, especially K^+^, Cl^−^, and malate (Mal), the total and free concentrations of Ca^2+^ and H^+^ and their buffering, and the corresponding ion fluxes through each transporter. For such modeling, it is sufficient to know the kinetic relationships that describe a process, even if the structural gene products are not. The same considerations apply to the molecular mechanisms behind their regulation. To understand how a system responds to physiological or experimental perturbation, the only relevant biology is encapsulated in how one model variable is connected to another. For example, we still do not know the relative contributions of the KAT1, KAT2, and KC1 subunits to the inward-rectifying K^+^ channels at the plasma membrane of Arabidopsis guard cells (Jeanguenin et al. 2011; Lefoulon 2021). Nevertheless, we know how the ensemble K^+^ current depends on voltage and how its amplitude depends on extracellular [K^+^], pH, and [Ca^2+^]_i_, and we can describe these dependencies in quantitative terms (Blatt 1990, 1992; Lemtiri-Chlieh and MacRobbie 1994; Grabov and Blatt 1997, 1999; Roelfsema and Prins 1997; Gajdanowicz et al. 2009). Such knowledge is sufficient to describe both dependencies and to model the current using a set of precise, mathematical equations. This approach introduces modules—“black boxes” with adjustable levels of kinetic detail—that may be expanded if, and when, a specific module becomes the focus of study; it also greatly reduces the computational overhead without a loss in predictive power (Endy and Brent 2001).

In general, the true value of any model rests in its ability not only to reproduce known behaviors but also to predict new ones that can be tested experimentally. Models built on a collection of elements generate system behavior through the interactions that arise between the elements; these interactions often lead to unexpected, or emergent, behaviors that could not be anticipated from knowledge of the elements in isolation. More often than not, these emergent behaviors become a focus for model testing and validation. Model and experiment are thus complementary tools (Stelling et al. 2004; van Riel 2006), each model representing a hypothesis to be discarded, validated, or refined by comparing model predictions with experimental results.

**Figure 5.**
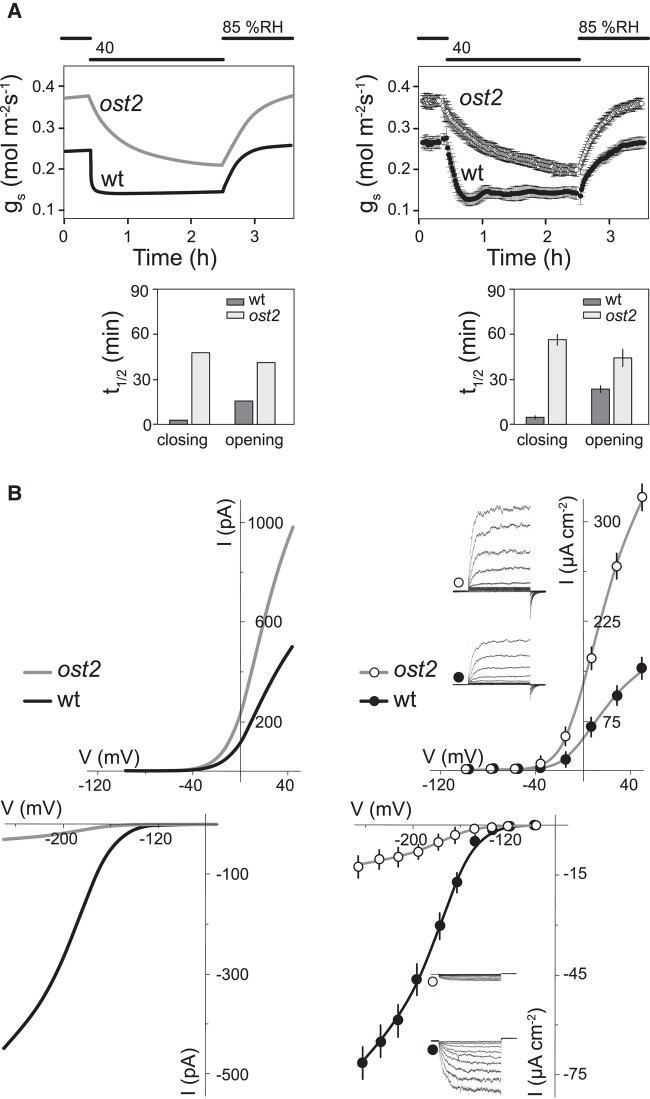
OnGuard correctly predicts slowed stomatal opening and closing in the *ost2* H^+^-ATPase mutant in response to steps in relative humidity. OnGuard modeling outputs (*left*) and experimental data (*right*) connect slowed stomatal reopening in the Arabidopsis *ost2* mutant with the greatly reduced activity of the inward-rectifying K^+^ channels when compared with wild-type Arabidopsis (wt). Modeling and experimental data consolidated and redrawn from Figs 3, 5, and 6 of Wang et al (2017). **A)** Stomatal conductances (g_s_) predicted (*left*) and measured (*right*) as means ± SE of 4 independent experiments. Relative humidity (%RH) steps indicated (*above*). Corresponding halftimes (*t*_1/2_) for closing and opening (*below*) were calculated by nonlinear least-squares fitting of model outputs and experimental data to a single exponential function. **B)** Current–voltage (IV) curves predicted (*left*) and recorded (*right*) under voltage clamp as means ± SE of 5 independent experiments. Steady-state currents shown are for the outward- (*above*) and inward-rectifying (*below*) K^+^ channels. *Insets (right)*: Representative current relaxations with voltage steps recorded under voltage clamp. Additional data and analysis (Wang et al. 2017) ascribes the enhanced outward-rectifying K^+^ current to a 0.3 unit rise in cytosolic pH and the reduced inward-rectifying K^+^ current to this pH rise and an increase in [Ca^2+^]_i_ to near 500 nM at rest. Slowed reopening in the *ost2* mutant was predicted as a direct consequence of reducing the inward K^+^ current by roughly 90%; the slowed closure was the predicted consequence of hyperpolarizing the membrane and raising pH, thereby suppressing activation of the R-type channels that engage the [Ca^2+^]_i_ and voltage oscillations needed to promote the outward-rectifying K^+^ channels and accelerate stomatal closure.

Models built on the original OnGuard platform (Chen et al. 2012; Hills et al. 2012) proved their predictive power (Table 4), among others, by explaining why guard cell [Ca^2+^]_i_ rises during the daytime, how Cl^−^ availability affects vacuolar Mal accumulation (Dodd et al. 2007; Chen et al. 2012), and how net Cl^−^ and Mal transport is directed to the vacuole during the day and out of the vacuole to the apoplast at night (Chen et al. 2012; Hills et al. 2012). These models predicted how the constitutively-active H^+^-ATPase *ost2* mutation affects Cl^−^ and Mal accumulation (Blatt et al. 2014) and slows stomatal opening as well as closing (Wang et al. 2017), as I noted earlier (Fig. 5). They also explained why stomata of the *slac1* mutant open as well as close slowly (Wang et al. 2012). These studies uncovered networks connecting the activity of the K^+^ channels with the anion channels and H^+^-ATPase. The model outputs include many other testable predictions, including the importance of gating of the outward-rectifying K^+^ channels as a target to accelerate stomatal kinetics (Blatt et al. 2014).

**Table 4.** Selected predictions arising from the OnGuard platform^a^

Prediction	Observations	Principal targets	Validated?^a^	Refs
**OnGuard1**				
*slac1*	KAT1 inhibition, slow opening [Ca^2+^]_i_ and pH_i_ increases	Ca^2+^ channel, H^+^–Cl^−^ symport, K^+^ channels	Yes	^b^
Elevated [Ca^2+^]_i_	Daytime rise in [Ca^2+^]_i_	H^+^-ATPase, Ca^2+^ channel, Ca^2+^-ATPases	Historic	^c^
Cl^−^ accumulation	preference for Cl^−^ over Mal accumulation with [Cl^−^]_o_	H^+^-Cl^−^ symport, VCl channel, VMal channel	Historic	^d^
Tonoplast voltage	Daytime depolarization	All tonoplast transporters	Untested	^e^
[Ca^2+^]_i_ oscillations	[Ca^2+^]_i_ oscillations accelerate closure, coupled with solute flux	H^+^-ATPase, Ca^2+^ channel, Ca^2+^-ATPases	Yes	^e^
**OnGuard2**				
*ost2*	Elevated pH_i_, slowed opening and closing, elevated [Ca^2+^]_i_	H^+^-ATPase, [Ca^2+^]_i_ increases, Ca^2+^ channel, VCa^2+^ channel, H^+^-Cl^−^ symport	Yes	^f^
*slac1* and water	No effect of *slac1* on VPD-driven stomatal movement		Yes	^f^
Mal and stress	preference for Mal retention under water/osmotic stress	VCl channel, SLAC1 channel	Historic	^d^
**OnGuard3**				
[Ca^2+^]_i_ oscillations	CO_2_-evoked [Ca^2+^]_i_ rise	VCa^2+^-ATPase, VCa_in_ channel	Historic	^g^
Carbon memory	Latency in stomatal closing	VCa^2+^-ATPase, VCa_in_ channel	Yes	^h^
CO_2_ “locking”	CO_2_ precedence over dark in stomatal closure	VCa_in_ channel	Yes	^j^
K^+^ channel engineering	K^+^ channel gating as a target to accelerate stomata	GORK K^+^ channel	Yes	^k^

^a^Listed are a small selection of the predictions arising from simulations with the OnGuard platform that are documented in the literature. Predictions are validated through previous publications (historic) or following the modeling (yes); other predictions remain to be examined (untested).

^b^ Wang et al. (2012).

^c^ Dodd et al. (2005, 2007) and Chen et al. (2012).

^d^ Allaway (1973), Van Kirk and Raschke (1977, 1978), Raschke and Schnabl (1978), and Chen et al. (2012).

^e^ Chen et al. (2012), Hills et al. (2012), and Wang et al. (2012).

^f^ Wang et al. (2017).

^g^ Webb et al. (1996), Grabov and Blatt (1997, 1998), Minguet-Parramona et al. (2016), and Jezek et al. (2021).

^h^ Jezek et al. (2021).

^j^ Blatt et al. (2022).

^k^ Blatt and Alvim (2022) and Horaruang et al. (2022).

With the second generation platform, we introduced foliar transpiration, connecting whole-plant water relations with water and solute transport in the guard cells (Wang et al. 2017). OnGuard2 incorporated vapor equilibration with water in the guard cell wall, harmonizing the concepts of stomatal vapor equilibration (Peak and Mott 2011) and of liquid water delivery to the mesophyll and guard cells (Buckley et al. 2003, 2017; Rockwell et al. 2014). Apoplastic solute and turgor “exchange” with the surrounding epidermal cells accommodated the opposing mechanical pressures of the epidermis and the visco-elastic properties of the guard cell wall (Jezek et al. 2019). Finally, with the third-generation platform, OnGuard3, we added CO_2_ diffusion from the atmosphere and its fixation through mesophyll photosynthesis (Jezek et al. 2021). Including CO_2_ diffusion allowed tests for the minimum set of targets necessary to explain CO_2_ action on stomatal aperture. It uncovered an emergent “carbon memory” in stomatal responsiveness and correctly predicted its impact on foliar gas exchange and water use efficiency as well as its dependence on endomembrane Ca^2+^ storage and release in the guard cell (Jezek et al. 2021). OnGuard3 has also proven instrumental in guiding the recent bioengineering of the GORK K^+^ channel to accelerate stomatal movement for gains in both water use efficiency and photosynthetic yield (Blatt and Alvim 2022; Horaruang et al. 2022).

What do we learn from OnGuard and, more generally, from mechanistic modeling of transport physiology? The foremost lesson, again, is just how poor intuition is as a guide to understanding the consequences of transporters operating in parallel across a common membrane and sharing substrates and products on either side. Simulations such as those possible with OnGuard are therefore keys to resolving the “decisions made” by stomata (Berry et al. 2010) and encoded within the network of transport entanglements. A second, and equally important, lesson is that a very large part of transport physiology is explicable through these entanglements, even without considering additional tiers of regulatory activity, whether of gene expression, vesicle trafficking, or post-translational protein modification. Of course, these additional tiers of control may be important for other reasons, for example in long-term adaptation or in fine-tuning transport. So, a third lesson is simply that modeling based on the mechanics intrinsic to transport is a good place to start and should be an early step in any systematic understanding physiology before refocusing research to address extrinsic regulatory processes.

In short, in recognizing the wealth of emergent behaviors, it becomes evident that much of stomatal physiology simply reflects “a spectrum of behaviors that emerge from the ionic fluxes that play across the guard cell membranes, their entanglement and balance with the metabolic activities of the guard cells” (Blatt et al. 2022). For those seeking fundamental knowledge, the challenge is to resolve these entanglements and the behaviors that they engender. Mechanism-based models are essential tools in this process. They are equally important, therefore, as practical guides in the effort towards rational biodesign of membrane transport going forward. If nothing else, the re-engineering of GORK gating (Blatt and Alvim 2022; Horaruang et al. 2022) is a clear example of how such knowledge can be put to use to improve whole-plant water use efficiency and photosynthetic carbon assimilation.

## Concluding remarks

The International Workshop on Plant Membrane Biology was held this past year in Taipei, delayed beyond the usual triannual occurrence by COVID. These meetings have a long history going back to 1968 when the first international gathering was held in Schloss Reinhardsbrunn, in what was then East Germany. The workshop title did not appear until the meeting in Liverpool in 1972, and the focus expanded from ion transport to membrane biology with the meeting in Monterey, California, in 1992. The science has similarly progressed from descriptive studies, analyses of the driving forces on ion flux, and questions around their energy dependencies to the quantitative kinetics of individual transporters, their molecular properties, regulation, and trafficking.

Our knowledge of transport protein structure is growing rapidly too (Table 1). Crystal structures for the Arabidopsis plasma membrane H^+^-ATPase AHA2 (Pedersen et al. 2007) and spinach aquaporin PIP2;1 (Tornroth-Horsefield et al. 2006) have been followed by detail of the structures, mechanics, and assembly of a bacterial SLAC1 channel homolog (Chen et al. 2010a) and more recently the Arabidopsis SLAC1 (Deng et al. 2021), the tonoplast aquaporin TIP2;1 (Kirscht et al. 2016) and TPC1 (Guo et al. 2016; Kintzer and Stroud 2016), the CNBD K^+^ channels KAT1, AKT1, and SKOR (Clark et al. 2020; Dickinson et al. 2022; Lu et al. 2022; Li et al. 2023), as well as the ALMT12 channel (Qin et al. 2022).

With its operation across a common membrane, ion transport is intrinsically modular and therefore well-suited to bioengineering. I have no doubt that the first tentative steps in this direction (Papanatsiou et al. 2019; Zhou et al. 2021; Horaruang et al. 2022) will soon become routine tools in research and its application, much as is becoming the case for synthetic photosynthesis (Miller et al. 2020). The means to these advances requires only that we combine kinetic and physiological data with molecular structural information of this kind, and that we use mechanism-based models to guide these efforts.

It is important, too, that we keep in mind from where these advances have come, and not only as a roadmap to past false starts and dead ends. The historical backdrop to research in plant membrane transport also informs on many opportunities that remain to be explored, including those set aside often enough because they appeared too difficult to pursue at the time. Recognizing and utilizing this history wisely is all the more urgent in light of the global imperatives of climate change and the need to husband our environment and its resources. Rodriguez-Martinez et al. (2022) make the case for solutions to come through molecular biology, but clearly the call applies to biological research generally and its potential for innovation.

Outstanding questions box

- A role for the slow-vacuolar cation channel TPC1 in plant physiology remains as obscure as it was 35 years on from its discovery.
- The identity of the plasma membrane Ca^2+^ channels that trigger [Ca^2+^]_i_ and voltage oscillations in guard cells and close stomata remains a mystery, as the current differs in biophysical characteristics from that of any cyclic nucleotide-gated channel identified to date.
- Similarly, the nature and identities remain obscure for the channels mediating endomembrane Ca^2+^ release and [Ca^2+^]_i_ elevations.
- Mechanistic models highlight the entanglements of ion transport, providing explanations for seemingly counterintuitive behaviors without a need for extrinsic regulation. A general challenge, then, is to establish what cannot be understood as a result of these entanglements and the behaviors that emerge from them.

## Dive Curated Terms

The following phenotypic, genotypic, and functional terms are of significance to the work described in this paper:

GORK Gramene: AT5G37500
GORK Araport: AT5G37500

SKOR Gramene: AT3G02850
SKOR Araport: AT3G02850

AKT1 Gramene: AT2G26650
AKT1 Araport: AT2G26650

ABP1 Gramene: AT4G02980
ABP1 Araport: AT4G02980

AHA1 Gramene: AT2G18960
AHA1 Araport: AT2G18960

AHA2 Gramene: AT4G30190
AHA2 Araport: AT4G30190

CNGC18 Gramene: AT5G14870
CNGC18 Araport: AT5G14870

TPK1 Gramene: AT1G02880
TPK1 Araport: AT1G02880

KAT1 Gramene: AT1G04710
KAT1 Araport: AT1G04710

KAT2 Gramene: At2g33150
KAT2 Araport: At2g33150

SLAH3 Gramene: AT5G24030
SLAH3 Araport: AT5G24030
